# Theoretical insights on helix repacking as the origin of P-glycoprotein promiscuity

**DOI:** 10.1038/s41598-020-66587-5

**Published:** 2020-06-17

**Authors:** Cátia A. Bonito, Ricardo J. Ferreira, Maria-José. U. Ferreira, Jean-Pierre Gillet, M. Natália D. S. Cordeiro, Daniel J. V. A. dos Santos

**Affiliations:** 1 LAQV@REQUIMTE, Department of Chemistry and Biochemistry, Faculty of Sciences, University of Porto, Rua do Campo Alegre, 4169-007 Porto, Portugal; 2 Science for Life Laboratory, Department of Cell and Molecular Biology, Uppsala University, 75124 Uppsala, Sweden; 3 Research Institute for Medicines (iMed.ULisboa), Faculty of Pharmacy, Universidade de Lisboa, Av. Prof. Gama Pinto, 1649-003 Lisboa, Portugal; 4 Laboratory of Molecular Cancer Biology, Molecular Physiology Research Unit-URPhyM, Namur Research Institute for Life Sciences (NARILIS), Faculty of Medicine, University of Namur, B-5000 Namur, Belgium

**Keywords:** Computational biophysics, Theoretical chemistry

## Abstract

P-glycoprotein (P-gp, ABCB1) overexpression is, currently, one of the most important multidrug resistance (MDR) mechanisms in tumor cells. Thus, modulating drug efflux by P-gp has become one of the most promising approaches to overcome MDR in cancer. Yet, more insights on the molecular basis of drug specificity and efflux-related signal transmission mechanism between the transmembrane domains (TMDs) and the nucleotide binding domains (NBDs) are needed to develop molecules with higher selectivity and efficacy. Starting from a murine P-gp crystallographic structure at the inward-facing conformation (PDB ID: 4Q9H), we evaluated the structural quality of the herein generated human P-gp homology model. This initial human P-gp model, in the presence of the “linker” and inserted in a suitable lipid bilayer, was refined through molecular dynamics simulations and thoroughly validated. The best human P-gp model was further used to study the effect of four single-point mutations located at the TMDs, experimentally related with changes in substrate specificity and drug-stimulated ATPase activity. Remarkably, each P-gp mutation is able to induce transmembrane α-helices (TMHs) repacking, affecting the drug-binding pocket volume and the drug-binding sites properties (e.g. volume, shape and polarity) finally compromising drug binding at the substrate binding sites. Furthermore, intracellular coupling helices (ICH) also play an important role since changes in the TMHs rearrangement are shown to have an impact in residue interactions at the ICH-NBD interfaces, suggesting that identified TMHs repacking affect TMD-NBD contacts and interfere with signal transmission from the TMDs to the NBDs.

## Introduction

**Figure 1:**
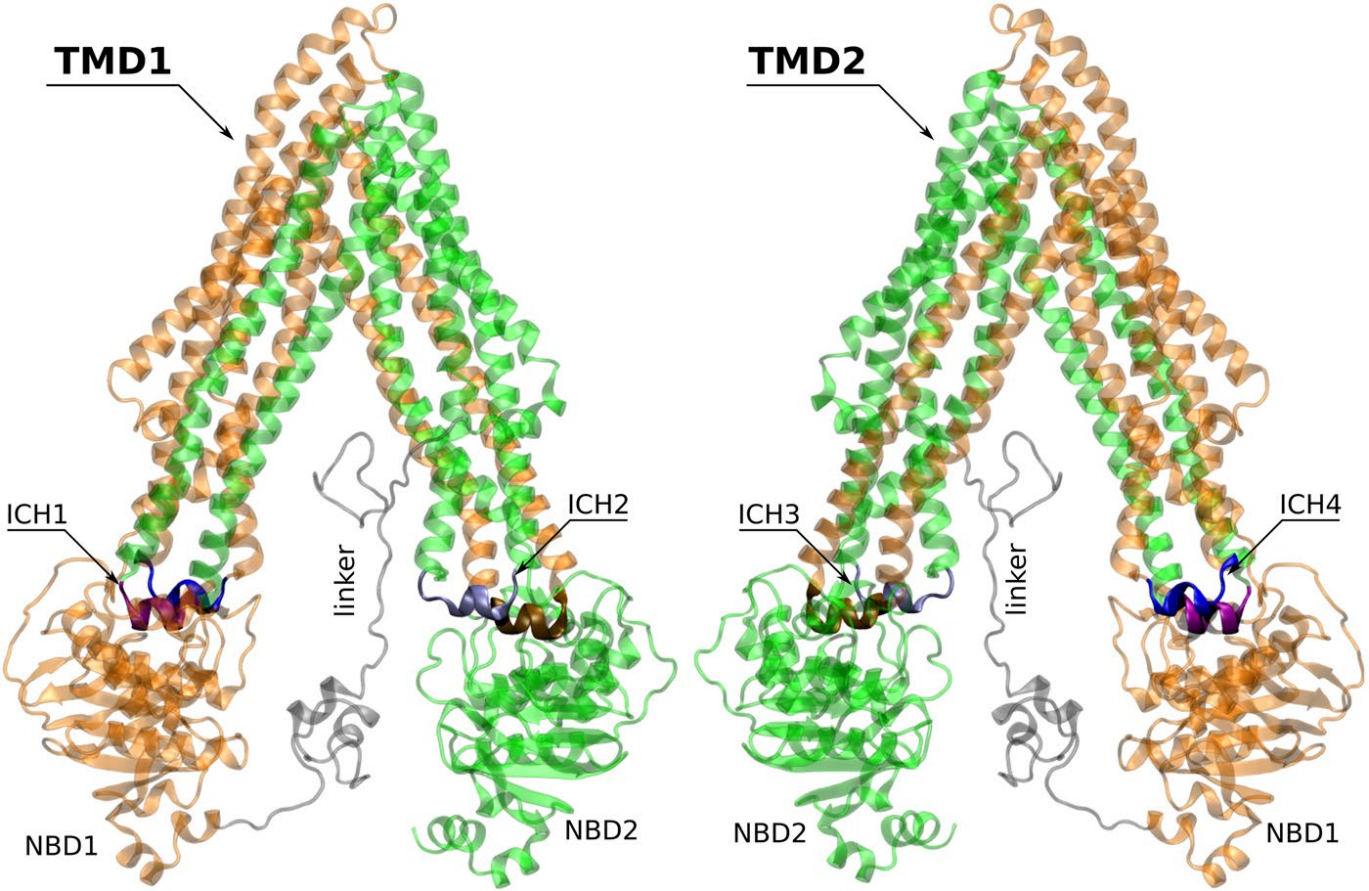
Structural representation of the human P-glycoprotein in an inward-facing conformation. The 12 transmembrane α-helices (TMHs) are divided into two transmembrane domains (TMD1; orange and TMD2; green), being physically linked to the respective nucleotide-binding domain (NBD) by coils bridging TMH6/NBD1 and TMH12/NBD2 and also by non-covalent interactions involving short intracellular coupling helices: ICH1 (purple)/ICH4 (blue) with NBD1 and ICH2 (silver)/ICH3 (brown) with NBD2. The DBP is a large cavity between both TMDs. Figures were created with MOE^133^ from the final human P-gp homology model.

Multidrug resistance (MDR) to anticancer drugs is, at the moment, a major contributor to chemotherapy failure^1^. In cancer, one of the most significant MDR mechanisms results from the overexpression of P-glycoprotein, a membrane efflux pump (P-gp, ABCB1)^2^. Thus, a deeper understanding on P-gp substrate specificity and efflux-related signal transmission mechanism remains crucial for the development of more potent and selective compounds able to modulate drug efflux^3^. P-glycoprotein exports a broad range of structurally unrelated compounds through an ATP-dependent mechanism^4^. P-gp is organized in two homologous functional units (N- and C-terminal halves) with a pseudo-2-fold symmetry. Each halve comprises one transmembrane domain (TMD), formed by six transmembrane α-helices (TMHs), and one cytoplasmic nucleotide-binding domain (NBD). Both N- and C-terminal halves are connected by a small peptide sequence (the “linker”; residues 627–688)^5,6^. The TMHs are directly linked to the respective NBD by the intracellular loops, through the functional TM helices 6 (NBD1) and 12 (NBD2) and non-covalently by short intracellular coupling helices (ICHs), located between the structural TMHs 2/3 (ICH1-NBD1), 4/5 (ICH2-NBD2), 8/9 (ICH3-NBD2) and 10/11 (ICH4-NBD1)^7,8^ (Fig. 1). These ICHs were found to be important for the maturation and folding of the P-gp transporter, being also involved in the signal transmission pathway between the TMDs and NBDs^9,10^.

The drug-binding pocket (DBP) is a large cavity formed by the TMHs of both N- and C-terminal P-gp halves and is capable of recognizing and accommodating several structurally distinct substrates. Each NBD contains the catalytic site for ATP binding and hydrolysis^11,12^. Several experimental^5,10,12–36^ and computational^6,37–47^ studies were performed to better understand the details of drug specificity and efflux. However, the information gathered from these experiments are often scarcely related and the mechanisms of drug recognition/specificity and efflux are still unsolved. Therefore, for additional insights on P-gp efflux mechanism, the study of P-gp variants that are experimentally related with altered drug-resistance phenotypes, and changes in the ATPase activity is an interesting approach.

The first mutation in the human P-gp was identified in the colchicine-selected multidrug-resistance cell line (KB-C1)^48^ and comprises a substitution of a glycine by a valine residue in position 185 (G185V; TMH3). This mutation is reported to confer increased resistance to colchicine, etoposide, doxorubicin and puromycin while decreasing the resistance to vinblastine, vincristine, taxol and actinomycin D. The same study reported, unexpectedly, a 3.6-fold decrease in colchicine binding and a 3.8–5.5-fold increase in vinblastine binding when compared with the WT transporter, leading the authors to conclude that G185V mostly affects the dissociation of drugs from P-gp rather than its initial association^49^. Later studies additionally concluded that the basal ATPase activity and the degree of activation by substrates was also increased in G185V, with only minor changes on nucleotide binding^50–52^. As it was also reported that G185V mutant is able to transport colchicine and etoposide in an energetically more efficient way, the residue at position 185 was identified as pivotal for transmitting conformational changes between the catalytic sites and colchicine-binding site^53^.

Similar alterations were observed in another glycine variant (G830V; TMH9), obtained by site-directed mutagenesis, in the presence of verapamil and colchicine^52,54^. Herein, increased resistance to colchicine (3.3-fold) and decreased resistance to actinomycin D (0.29-fold) was reported but no significant change on vinblastine resistance was observed when compared with the WT protein^52^. Its verapamil-stimulated ATPase activity was found to be only slightly increased (1.4–1.7-fold), but no change in the vinblastine-stimulated ATPase activity was reported. Nonetheless, the pattern of drug-stimulated ATPase correlated well with the relative drug-resistance profiles in transfected cells^54^. No data on basal ATPase activity was provided.

Likewise, mutations involving the phenylalanine residue at position 978 were also identified to play an important functional role in P-gp. By mutating F978 to an alanine (F978A) a significant alteration of the drug resistant pattern was observed, conferring little or no resistant to either colchicine or doxorubicin but not changing the resistance to vinblastine or actinomycin D. This was further suggested to be due to a defect in the mutant protein, being unable to transport colchicine and having a reduced capability to transport vinblastine, even with an increased expression of the transporter. Quite interestingly, when testing other substitutions (F978S, F978L or F978Y), only the latter restored similar drug-resistance profiles for all tested molecules^55^, but nonetheless azidopine labeling was indistinguishable from WT protein. Interestingly, including the previously characterized G185V together with F978A/S was also unsuccessful in fully restoring the ability of conferring resistance to colchicine^55^. In addition, little or no drug-stimulated ATPase activity by colchicine or verapamil was reported, being suggested that F978A mutants have either a decreased affinity for substrates and/or an impairment in coupling drug binding to ATPase activity^54^. Recent studies, using a F978C mutant, additionally showed complete absence of stimulation of ATP hydrolysis by several drugs, proposing that F978 residue is part of a common translocation route, crucial for the propagation of conformational changes following ATP hydrolysis and important for the translocation process of high-affinity substrates^56^. Again, no data was reported on basal ATPase activity for this mutant.

Finally, the deletion of a phenylalanine residue at position 335 (ΔF335; TMH6) was reported in a multidrug-resistant variant cell line (DxP), by co-selection with doxorubicin and the cyclosporine D analogue, valspodar (PSC833), a potent P-gp modulator^57–59^. The transfected cells harboring the ΔF335 P-gp variant showed to be resistant to many chemotherapeutic drugs such as doxorubicin, daunorubicin, etoposide and paclitaxel. On the other hand, this variant conferred little resistance to vincristine, vinblastine and actinomycin D as well as a decreased ability to bind or transport cyclosporine, valspodar, vinblastine, actinomycin D and rhodamine-123, suggesting that the region surrounding the F335 residue is an important binding site for these compounds^59,60^. An enhancement of photoaffinity binding by [^125^I]-iodoarylazidoprazosin in the presence of verapamil or PSC833^59^ and a decrease of [^3^H]-cyclosporine binding^60^ was also referred. Moreover, by deleting the F335 residue a 2-fold increase in the basal ATPase was observed but, when in the presence of drugs, only verapamil-stimulated ATPase activity occured^60^. Interestingly, this mutant also presented a substantial decrease of 8-[*α*-^32^P]azido-ATP labeling when compared with the WT P-gp, which lead the authors to suggest that the ATPase activity of the mutant protein may depend on the helix conformation defined by F335^60^.

In this work, a human P-gp homology model in the apo inward-facing state conformation was generated based on the most recent murine P-gp crystallographic structure available in the beginning of our study (PDB IDs: 4Q9H)^61^. The P-gp model was refined through molecular dynamics (MD) simulations and validated using several approaches. The final human P-gp model was further used to understand the possible structural impact of the mutations described above on P-gp architecture/organization.

## Material and Methods

### Human P-gp homology modeling

The FASTA sequence of human P-gp was obtained from the Universal Protein Resource (UNIPROT) under the code P08183 (www.uniprot.org). The murine P-gp crystallographic structure (PDB IDs: 4Q9H), used as template, was retrieved from the Protein Data Bank (PDB; www.rcsb.org)^62,63^. Both the murine P-gp crystallographic structure and the FASTA sequence human P-gp were loaded into the MOE software and aligned by their sequence. The “linker” secondary structure, missing in all P-gp crystallographic structures so far, was obtained from a previously equilibrated murine P-gp^39^ and used as template for modeling the respective sequence (A627-A688). Herein, the insertion of the “linker” in the novel structures was achieved by aligning the human P-gp homology model with the crystallographic template, followed by an “override” of the gap in the considered structure in which the murine P-gp linker was used as template to obtain the human homologue. Afterwards, 25 mainchain models sampling 25 sidechain orientations were performed, producing 625 models for the human P-gp structure in MOE (force field parameters used by default). The model with lowest total potential energy was selected, protonated using the Protonate 3D module^64^ and exported as PDB file to be used by GROMACS version 5.0.7^65–67^. This way, an initial human P-gp homology model in an inward-facing conformation incorporating the “linker” obtained from a previously equilibrated murine P-gp^39^, was obtained.

### Construction of the protein membrane system

The topology of the human P-gp model was generated according with the GROMOS96 54a7 force field. A previous equilibrated 1-palmitoyl-2-oleoyl-phosphatidylcholine (POPC) membrane patch^39^ was used with the lipid parameterization by Poger *et al*.^68,69^. The human P-gp homology model was inserted into a lipid bilayer (longer P-gp axis perpendicular to the *xy* membrane plane) to match the hydrophobic thickness of TMDs and membrane. The relative position of the membrane was obtained from the Orientations of Proteins in Membranes (OPM) database^70^ (http://opm.phar.umich.edu) and protein insertion was achieved through the *g_membed*^71,72^ module in GROMACS.

The protein embedded into the lipid bilayer was centered in a simulation box with dimensions *xyz* of 12.76 × 12.76 × 16.50 nm^3^ and periodic boundary conditions (PBC). Finally, the system was solvated and neutralized with an adequate number of water molecules and counterions using other GROMACS’ modules.

### Molecular Dynamics: equilibration and production run

Firstly, an energy minimization run comprising the whole system was applied using the steepest descent method. Then, the temperature of the membrane system (303 K) was equilibrated for 10 ps in the *NVT* ensemble, spatially restraining all protein´s heavy atoms. Following, the POPC lipid bilayer was allowed to correctly adjust to the protein interface through a 20 ns *NpT* run, still keeping the protein’s heavy atoms restrained. Finally, three sequential 500 ps *NpT* runs were performed to progressively remove the protein’s heavy atoms spatial restriction (mainchain, backbone and alpha-carbons, respectively). This system was the starting point for a 200 ns fully unrestrained *NpT* production run (Fig. S1, Supporting Information).

### Model quality assessment

The stability of the P-gp model was monitored along the MD run through the evolution of the root mean square deviation (RMSD) of the Cα atoms, visual inspection and the MolProbity^73,74^ evaluation server. After 200 ns of simulation time, more exhaustive evaluations were performed, through additional servers namely ERRAT^75^, PROCHECK^76,77^ and SwissModel Structure assessment tool^78–81^. Moreover, the stability and quality of the human P-gp model were also assessed considering the Ramachandran plot^82^ and by checking for correlations between molecular docking and experimental data. The evaluation of the recently published human cryo-EM P-gp structure was also performed, for comparison purposes.

### Construction of the human P-gp mutated structures and systems

From the final refined human P-gp homology model, four human P-gp variants (G185V, G830V, F978A and ∆F335) experimentally linked with changes in efflux and substrate specificity were built using MOE. Each P-gp variant was then embedded into a POPC membrane, water solvated and charge neutralized as described above. Energy minimization runs comprising the whole system were applied followed by a 10 ps *NVT* run at 303 K by spatially restraining all protein’s heavy atoms. Fully unrestrained *NpT* runs followed for 100 ns (Fig. S1). After 50 ns of simulation time, two system replicates were obtained for all P-gp variant systems, each one simulated for another 50 ns by randomly generating initial velocities, assigned from the correct temperature dependent Maxwell-Boltzman distribution, and starting with the final configuration obtained at the end of the first 50 ns. This way, for each P-gp variant, three replica systems were therefore simulated in a total of 200 ns of simulation time.

### Simulation parameters

All *NVT* equilibration runs were performed at 303 K using the Velocity-rescale (V-rescale)^83^ thermostat. The Nosé-Hoover^84,85^ thermostat and the Parrinello-Rahman^86^ barostat for temperature (303 K) and pressure (1 bar), respectively, were applied in all *NpT* runs. Due to the presence of membranes, pressure equilibration was achieved through a semi-isotropic pressure coupling, with the systems’ compressibility set to 4.5 × 10^−5^ bar^−1^. All bond lengths were constrained using the LINCS^87,88^ and SETTLE^89^ (for water molecules) algorithms. The Particle Mesh Ewald (PME) with cubic interpolation^90,91^ was employed, with a cut-off radius of 12 Å for both electrostatic and van der Waals interactions and an FFT grid spacing of 0.16 for long range electrostatics. Group-based and Verlet^92^ cut-off schemes were applied for the calculation of non-bonded interactions on CPU or GPU, respectively.

### Structural analysis of the human P-gp variants

To evaluate the impact of each mutation in the volume of the internal DBP (only considering the transmembrane helical bundle buried within the membrane) and to allow a fast comparison between WT and variants, the DBP was estimated as the sum of the volumes of all water molecules found inside this cavity through in-house python scripts. To assess the effect of the mutations on TMDs rearrangement, the TM bundle was analyzed through the *g_bundle* module available in GROMACS. The total number of contacts between the ICHs residues and the respective NBD were calculated using the *g_hbond*^93^ module and the contact frequencies were estimated by the *g_contacts*^94^ module. Furthermore, the EPOS^BP^ ^95,96^ software (default parameters) was used to characterize the DBSs found within the DBP of the human WT P-gp model and variants. The top-ranked docking poses of each molecule in each zone were then overlapped with the cavity search results identifying, this way, lining atoms (within a distance of 5 Å from the pocket probes) and calculating mean pocket volumes and polarities (ratio of the sum of N, O, and S atoms to the sum of N, O, S, and C atoms). Visual inspections were performed with VMD^97^ and MOE software. All analysis described above were performed using the last 50 ns of each simulation.

### Docking studies

Molecular docking was performed using the final human WT P-gp model and the generated variants (G185V, G830V, F987A and ΔF335). The chosen databases comprised P-gp substrates (*N* = 33), probes (*N* = 7) and modulators (*N* = 19), previously used in docking studies with our refined murine P-gp structure^39^. The ligands binding location was defined by a docking box comprising the whole internal cavity identified by Aller *et al*.^5^, with dimensions *xyz* of 32.25 × 26.25 × 37.50 Å^3^, and centered at the DBP (*xy* corresponds to the membrane plane). Due to the large search volume (over 30.000 Å^3^), Vina’s ‘exhaustiveness’ parameter was manually set to 50 and twenty docking poses were generated. Visual inspection of the best ranked docking poses was made in MOE to allow the identification of individual docking zones.

## Results and Discussion

### Human P-gp homology model development

Considering the high sequence identity and similarity with the human P-gp efflux pump (87% and 94%, respectively)^98^, a human P-gp homology model was obtained, using the murine P-gp crystallographic structure of 2015 (PDB ID: 4Q9H) as template.

The murine P-gp crystallographic structure was chosen based on: i) it was the most recently published murine P-gp crystallographic structure in the beginning of our study, ii) it shows improvements in the resolution of several TMHs, ICH1 and some extracellular loops in respect to previous structures, stressing the quality of the starting template to the development of a reliable human P-gp model, and iii) oppositely to the recently published human P-gp structure^99^, it was obtained without any ligands or antibody complexes that could have any influence on the native arrangement of the transmembrane helical domains.

After 14 ns of simulation time, and unlike the refined murine P-gp structure^39^, a shift of the linker’s upper loop downwards was observed in our human P-gp model (v1 model). Thus, to assess the correct position of the “linker” in the human P-pg model, two snapshots retrieved at 8 ns (v2 model) and 14 ns (v3 model) of simulation time were the starting points for two additional MD runs (Fig. S1).

At the end of 200 ns MD simulations, a shift of the linker’s upper loop downwards was also observed in both v2 and v3 models confirming the possibility that this new position acquired by the “linker” structure is favored in our model. This result also demonstrates the high flexibility of the “linker” region offering a plausible explanation for its absence in all crystallographic structures so far. Although an adjustment of the NBDs was observed in all P-gp models, both v1 and v2 models also revealed distortions in the “linker” secondary structure, namely through the formation of an α-helix in its middle coil. Additionally, a kink in the TMHs 6, 10 and 12 was found in both models compromising the DBP bottom and the portals. Together with the low scores obtained in the evaluation servers (Table S1), the identification of structural alterations were the underlying reasons leading to the rejection of both v1 and v2 models. However, a stable human P-gp model was obtained after 200 ns of simulation time, although with a POPC lipid molecule located in the portal 4/6 (v3a model), in agreement with the computational findings of Tajkhorshid and colleagues^100^. Interestingly, no secondary structure in the middle coil of the linker structure was found in this model. As it is experimentally known that some lipids play an important role in the stability and function of protein membranes including P-gp^101–103^, it was important to assess the impact of the lipid molecule in the protein stability. Therefore, two new system snapshots were retrieved from the v3a model at 200 ns of simulation time as starting points for two additional 200 ns MD runs (in a total of 400 ns of each system), where the POPC molecule was removed from the system (v3b) or moved into the lipid bilayer (v3c) (Fig. S1).

### Human P-gp homology model validation

Although lower scores were observed in the initial human P-gp model obtained with MOE, significant structural improvements were achieved in the refined models, after molecular dynamics (MD) simulations (Table S1). Although the SwissModel online server still utilizes an algorithm that is not adequate for evaluating membrane proteins^104,105^, all our structures scored above −5.0, a solid indicator on the good quality of the herein developed models^104^.

Another important tool to assess the structural quality of a given structure is through the analysis of the Ramachandran plots of each v3 P-gp model (Fig. S2). Substantial improvements were observed in the refined P-gp models with only 16 (v3a), and 19 (v3b) and 18 (v3c) outliers, respectively versus 34 outliers found in the initial P-gp homology model. The outliers were found mainly in coils, being the only exceptions A348 residue, located at the TMH6 (v3a and v3b), and the F359 (TMH11) and N81 (TMH1) residues in the v3c model.

The RMSD evolution of the P-gp models during MD simulations (Fig. S3) showed that any considerable changes in protein’s conformation occurred during the first 200 ns of simulation time. Moreover, a comparison between the refined v3 models showed that all models were stable during the last 200 ns of MD runs with the POPC molecule initially found in the portal 4/6 (v3a model) not affecting the protein stability. However, visual inspection of the three P-gp models revealed differences in the TMHs spatial positions when compared to the starting MD structures (200 ns). While no changes were observed in both v3b and v3c models, the POPC molecule found in the portal 4/6 in the v3a model produced deviations in the spatial arrangement of the TMHs 4 and 6. Moreover, in the v3b model (obtained by removing the POPC molecule from the system’s topology), changes in the positions of most TMHs were observed, including helices 4/6 and 10/12 forming both DBP portals. When the POPC was moved into the lipid bilayer in the v3c model, only slight differences in the spatial position of the TM helices 1, 3 and 4 were observed (*Supporting PDB file*).

All v3 models were also validated using docking studies to assess the number of DBSs within the DBP. The molecules tested were clustered in substrates, probes and modulators, in agreement with experimental^106,107^ and *in silico* data^40^. Three DBSs were found inside the DBP in the v3c model, in agreement with the previously refined murine P-gp structure^39^ and experimental data^5,20,25,108^, while in both v3a and v3b models the three DBSs were found to be less defined (data not shown).

Altogether, our results showed significant improvements in the quality of human P-gp models after MD simulations indicating that the presence of the “linker” and lipid bilayer are undoubtedly important for the stability of the transporter, in agreement with other studies^39,40,104^.

**Figure 2:**
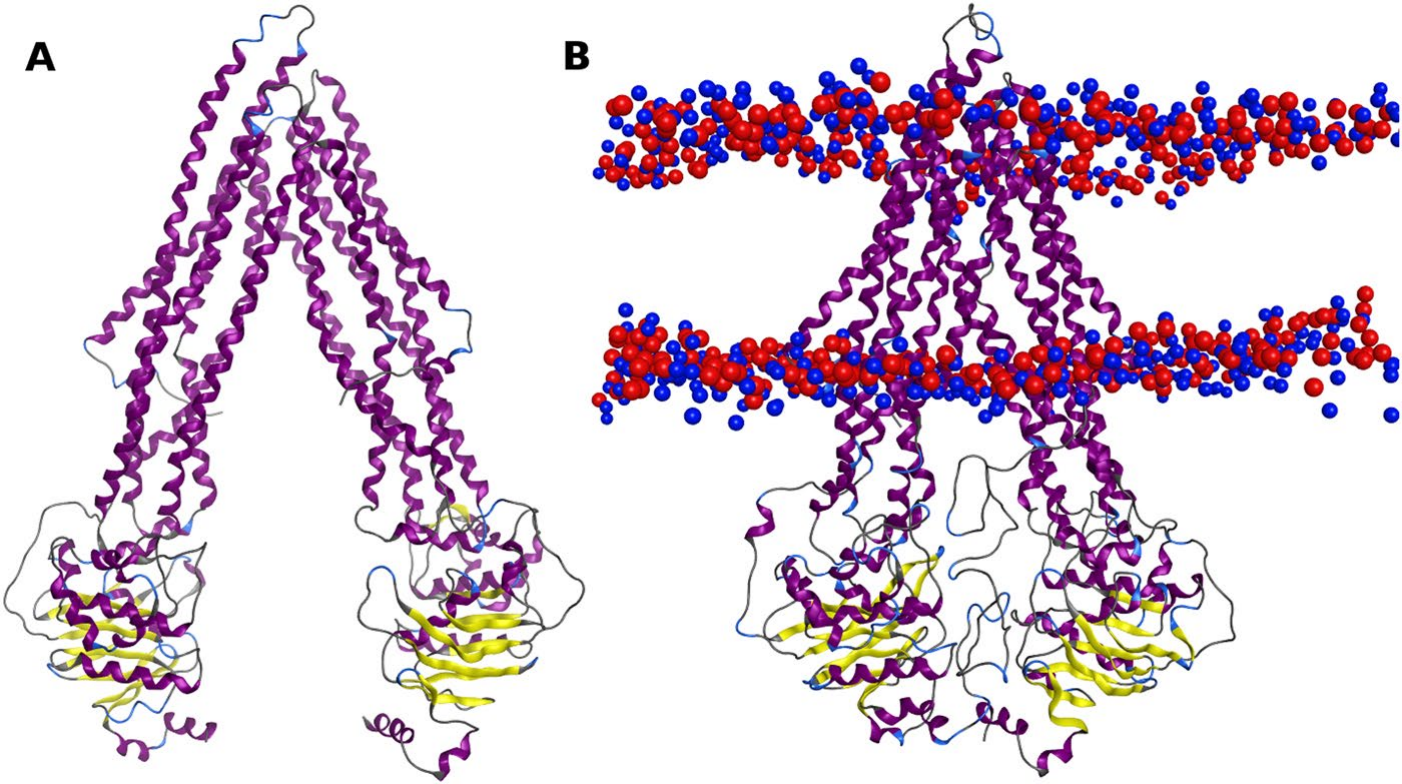
(**A**) Representation of the murine P-gp crystallographic structure (PDB ID: 4Q9H) used as template vs (**B**) human WT P-gp model (v3c) in the presence of the “linker” and a POPC membrane. The lipid bilayer boundaries are represented through the phosphate (red) and nitrogen (blue) atoms of the lipid headgroups.

The analysis of the online evaluation servers, Ramachandran plot and RMSD did not reveal significant changes among the refined human P-gp models. However, as differences in the TMHs spatial position and number of DBSs within the DBP were found in both v3a and v3b models, the v3c model was considered the most stable and suitable human P-gp homology model to study the human P-gp variants. This model will be named as wild-type (WT) (Fig. 2). To compare the data gathered from the analysis of P-gp variants with the human WT model, v3c MD run was extended from 400 ns to 500 ns. This way, after a total of 700 ns of simulation time, a stable human P-gp model with high quality and robustness was obtained.

During the development of this work, the first human P-gp cryo-EM structure in a nucleotide-free inward-facing conformation was made available (PDB ID: 6QEX)^99^. According to the online servers (Table S1), our human WT P-gp model (v3c) presents better scores than the recently published 6QEX structure, indicating that the quality of our P-gp model is not lower than the quality of the 6QEX structure. Additionally, in the recently available QMEANBrane^109^ server our human WT model also displayed similar local scores (0.746; WT vs 0.783; 6QEX) but more favorable membrane insertion energy (Fig. S4). Structural alignment between the human WT P-gp model and 6QEX structure showed similar spatial positions of the transmembrane and cytoplasmic domains when compared with the initial template (Figs. S5 and S6). Nevertheless, significant differences in the secondary structure of the TMH4 (S237–A248) and TMH10 (S880-K885) were found between our P-gp model and 6QEX structure. When comparing the RMSD of each TMH (Table S2), TMH4 and TMH10 are the helices that present higher RMSD values between the WT model and 6QEX. Interestingly, a chimeric human-mouse P-gp cryo-EM structure (PDB ID: 6FN4)^110^ presents similar alterations at the same regions of TMHs 4 and 10 and high RMSD values when compared to the human WT P-gp model.

In order to understand the nature of the TMH4 and TMH10 alterations in the human P-gp cryo-EM structure, four mouse P-gp crystallographic structures in the *apo* state inward-facing conformation (PDB IDs: 4M1M, 4Q9H, 5KPI^111^ and 6GDI^112^) and in the presence of ligands at the DBP (PDB IDs: 4M2S^113^ and 4Q9L^61^) were used for comparison. Visual inspection showed that both TMH4 and 10 are continuous α-helices in all *apo* and *holo* P-gp crystallographic structures considered, as observed in our human WT P-gp model. Moreover, Kim *et al*.^114^ recently published a human P-gp cryo-EM structure in the ATP-bound, outward-facing conformation (PDB ID: 6C0V), demonstrating that the TMHs 4 and 10 must be continuous α-helices to ensure the correct closure of the cytoplasmic pore when shifting from the inward to outward-facing conformation upon NBD dimerization. Furthermore, structural alignment between each P-gp crystallographic structure and the human WT P-gp model also revealed lower RMSD values for both TM helices 4 and 10, in contrast with what was observed in the 6EQX structure (Table S2). As P-gp is a highly flexible protein, we cannot exclude that the recently cryo-EM structure captured some degree of dynamic transitions between several conformations of TM helices 4 and 10, but the above results also imply that v3c is a robust and reliable human WT P-gp model that can be further used in mechanistic and drug discovery studies. The comparison with the experimental data retrieved from the P-gp crystallographic structures indicate that our human WT model, although preserving the secondary structure of the TMDs, retains enough flexibility in the TM helices 4 and 10, thus being comparable to that observed in the human 6QEX structure.

### Structural analysis of the human P-gp variants

To gain additional insights on drug specificity and efflux-related signal transmission mechanism, the structural impact of four P-gp mutations (G185V^48–54^, G830V^53,55^, F978A^53,55,56^ and ∆F335^57–60^), experimentally linked with changes in efflux and substrate specificity were analyzed. The mutations were selected according to their location within the DBP namely, at the substrate binding sites (SBSs) H and R sites (G185V and G830V, respectively) and at the modulator binding site (M-site) (F978A and ∆F335) (Fig. S7). Herein, while both H and R sites were initially characterized by their interaction with Hoechst 33342^108^ or Rhodamine-123^35^, respectively, the modulator M site was identified from the localization of the co-crystallized ligands QZ-SSS and QZ-RRR in the first crystallographic structure of murine P-gp^5^. Regarding SBSs, both were later characterized by molecular docking^40^ and experimentally confirmed by electron microscopy^112^.

One of the properties that can be altered by mutations in the transmembrane region of P-gp pump is the volume of the internal cavity. An estimation of the probability distribution function of the DBP volumes P (V) (Fig. S8) in the human WT P-gp model and variants showed that all mutations induced a reduction of the DBP volume, more pronounced in the F978A variant. Nevertheless, while G830V and F978A variants still sampled a wide range of DBP volumes during MD simulations, as observed in WT, G185V and ∆F335 variants showed narrower volume distributions suggesting a different structural cohesion of the transmembrane domains.

Thus, to investigate this hypothesis, the TMDs arrangement was analyzed through *g_bundle* and compared to the human WT P-gp model. Accordingly, this tool gives information about the bundle of axes (e.g. TMHs) such as the distance, length, and z-shift of the axis mid-points with respect to the average center of all axes as well as the total, lateral and radial tilt with respect to the average axis (*xy* corresponds to the membrane plane and *z* to the longest protein axis). The statistically significant changes in the bundle parameters are summarized in the Supporting Information (Tables S3–S8 and Figs. S9–S20).

Overall, the results show distinguishable changes in the TMHs repacking in all P-gp variants, including the helices where the respective mutation is located (Supporting PDB file). Moreover, all mutations showed significant changes in the bundle parameters of the TMHs 4/6 and 10/12 that form the DBP portals and in the “crossing helices” 4/5 and 10/11, that directly linked the TMD1 to NBD2 and TMD2 to NBD1. However, significant differences in the TMHs repacking between the mutations lying at the SBSs (G185V and G830V) and the mutations located at the M-site (F978A and ∆F335) were found.

Although being located at opposite helices (Fig. S7), the G185V and G830V mutations surprisingly showed similar structural changes in the TMHs rearrangement involving both TMDs. However, G830V mutation located at the TMH9 in the C-terminal P-gp halve seemed to have a stronger impact in the TMHs reorientation when compared to the G185V mutation in the TMH3 (Tables S5–S7). On the other hand, mutations at the M-site showed a completely distinct behavior. While the F978A mutation (TMH12) seemed to preferentially affect the TMHs rearrangement of the C-terminal P-gp halve where the mutation is located (Tables S4–S8), the deletion of F335 residue (∆F335, TMH6) apparently did not have a severe impact in the TMHs rearrangement as could be initially expected (Supporting PDB file).

Altogether, the analysis of the helical bundle indicates that (i) all P-gp mutations affect the TMHs repacking including the TMHs that form the DBP portals, which may compromise the access of drugs to the internal cavity, (ii) all P-gp mutations affect the bundle of the “crossing helices” 4/5 and 10/11, described as important helices in the NBD dimerization process upon ATP binding^114^ and (iii) mutations located at the SBSs have a different impact on helical repacking than those located at the M-site. Finally, as a result of the TMHs reorientation, all the selected mutations induce a reduction of the DBP volume in relation to WT (Fig. S6), but while both glycine and ΔF335 mutations induce a slight decreased in the pocket volume, the partial repacking caused by the F978A mutation may explain the severe reduction of the DBP volume found in this variant.

### Interactions between coupling helices and nucleotide-binding domains

Since the selected P-gp variants are experimentally linked with altered drug-resistance profiles and changes in either the basal or drug-stimulated ATPase activity, the residue interactions at the ICH-NBD interfaces thought to be involved in signal transmission and efflux-related conformational changes^9,10,26,45,115^ were evaluated and compared to the human WT P-gp model. The total number of contacts was estimated using the *g_hbond* module and is depicted in *Supporting Information* (Figs. S21 and S22).

For both glycine variants, only at ICH3-NBD2 interface a significant decrease in the total number of contacts was observed. Oppositely, both M-site variants displayed a distinct behavior. While the F978A variant showed a decrease in the total number of contacts at the ICH3-NBD2 interface (similar to the glycine mutants), ΔF335 showed significant changes in the total number of contacts in three of the four interfaces, namely ICH2-NBD2, ICH3-NBD2 and ICH4-NBD1. As all mutants seemed to induce changes in the ICH-NBD total number of contacts, we further identified which residue pairs were involved. Mean residue-residue contact frequencies ≥ 0.5 and variations above 10% were considered significant and are summarized at *Supporting Information* (Table S10). For clarity purposes, each ICH-NBD will be analyzed separately in the following section.

Concerning the ICH1-NBD1 interface, all P-gp variants showed an increase of the mean contact frequencies between I160 (ICH1) and L443 (Walker A, NBD1), both located in regions identified to be involved in ATP binding^26,116,117^. Oppositely, only in F978A and ΔF335 a decrease in contact frequencies involving D164 (ICH1) and R404 (NBD1) was observed. Quite interestingly, D164 was also reported to be part of an extensive interaction surface between the TMDs and NBDs, with D164C mutants additionally revealing lower cell surface expression^118^. Regarding the ICH1-NBD1 hydrogen bond network, a significant decrease in the hydrogen-bond lifetimes (life, Table S10) was also observed for all mutants.

The other interface at this nucleotide-binding domain is the ICH4-NBD1. Herein, the most affected residue pairs were R905–S434/Q438/Q441, with a greater decrease of contact frequencies in both glycine mutants; V908/R467 (increased in all mutants); and S905/Y401 and S909–Q441/R467/V472, mainly increased in the F978A and ΔF335 mutants. Again, mutational studies implied both S905 and S909 in the activation and ATPase stimulation when in the presence of drugs and/or lipids^26,30^. Interestingly, and specifically concerning the ΔF335, new contacts between V907/F480, L910/R547, E913/R464 and Q914/R464 explain the increase in the total number of contacts reported above. Regarding the hydrogen bond network between the ICH4 and NBD1 residues, a significant decrease in the average HB number, lifetime and energy of HB formation was observed only in both glycine mutants (Table S10).

At the opposite NBD, all mutations seem to induce a general decrease in the overall contacts frequencies, with the ICH2 residues I265 and F267, involved in P-gp maturation and activity^10,32^ and the NBD2 residue R1110, being the most affected ones. However, this decrease is partially mitigated by a reinforcement of the HB network in all mutations and, specifically for the ΔF335, through new contacts between F267 (ICH2) and G1134/R1188/A1189/R1192 (NBD2) or G269 (ICH2) and N1136 (NBD2) (Table S10). Finally, and regarding residue F1086, identified through *in vitro* studies as important for coupling of ATP binding to conformational changes in the TMDs^10^, almost all contacts frequencies decrease except when paired with R262 (G185V and F978A) or I265 (ΔF335).

Finally, for the ICH3-NBD2 interface, all variants showed, in general, a negative variation in the contact frequencies, most particularly between V801/S802 (ICH3) and Y1087 (NBD2), thought to play an important role in P-gp activity and assembly^115^. In the same way, all mutations induced a decrease of the contact frequencies between the D805 (ICH3), an important residue thought to be involved in the TMD-NBD communication^119^, and Y1044, located at the A-loop of NBD2.

Altogether, these results indicate that although mutations in the TMDs of the human P-gp affect directly the transmembrane region with changes in the DBP volume and DBSs features, they also induce changes in the residue interactions at the ICH-NBD signal transmission interfaces involved in the TMD-NBD communication. However, a comparison among P-gp variants showed that both mutations at the SBSs (G185V and G830V) induce identical changes in the total number of contacts between the ICH and NBD residues, while the impact of the M-site mutations (F978A and ΔF335) seems to be dependent of the TMH where the mutation is located, as observed in the helical bundle. Finally, the analysis of the mean contact frequencies in the human WT P-gp model and its variants, identified some residue pairs potentially involved in TMD-NBD communication, indicating an interaction network between the ICHs and NBDs residues, including those that directly interact with ATP, in agreement with several other studies^10,26,45,119,120^.

## Docking Results

### Identification of Drug-Binding Sites in the human P-gp model

Based on the docking poses of known P-gp substrates, probes and modulators, Ferreira and co-workers^40^ defined the location of the DBSs within the DBP (firstly identified by Shapiro *et al*.^20,24,25^ and Aller *et al*.^5^) using a refined murine P-gp structure^39^. To acquire a deeper knowledge on P-gp substrate binding, docking studies targeting the DBP of the human P-g WT model were undertaken. As twenty docking poses were generated per molecule, to simplify the results only the top-ranked binding energies (ΔG) at each DBS will be compared to the data obtained from previous studies^40^.

Overall, most of the molecules tested bound at the three DBSs found within the DBP of the human WT model (Table S9), as observed in the murine P-gp structure. Nevertheless, higher number of molecules interacted with the H-site in respect to the refined murine P-gp. However, considering the standard error reported for VINA (2.85 kcal·mol^−1^)^121^, no conclusions could be made regarding the affinity of these compounds since they have similar ΔG among the DBSs within the DBP of the WT model and similar ΔG than those reported in the refined murine P-gp structure.

**Figure 3:**
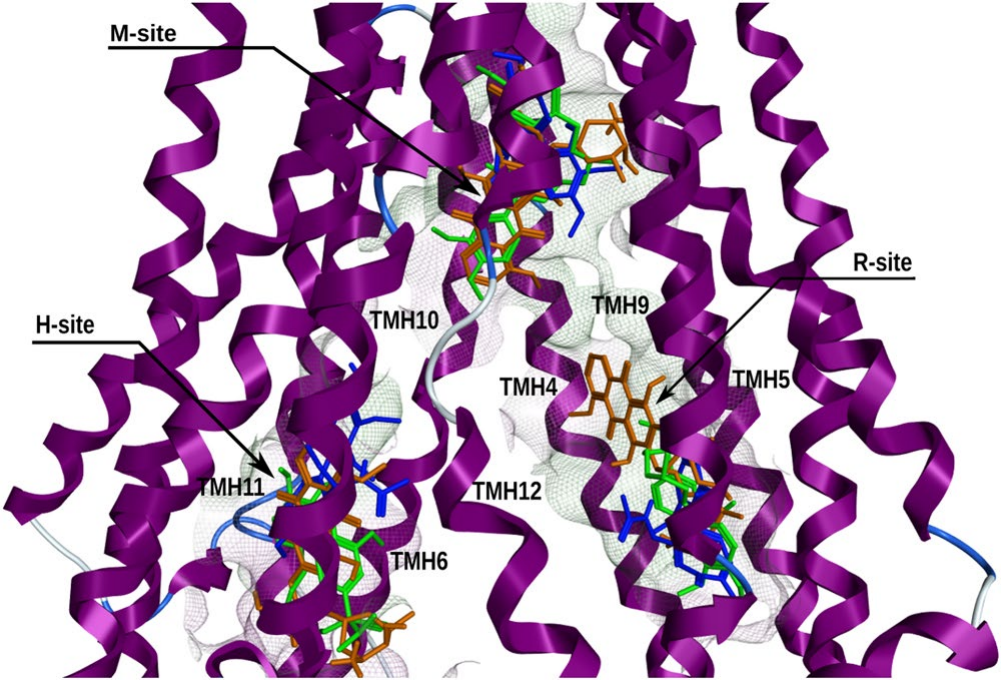
Representation of the DBSs found within the DBP of the human WT P-gp model. The three DBSs are defined by the best-ranked docking poses at each binding cavity of well-known P-gp substrates and modulators e.g. verapamil (green), doxorubicin (dark orange) and colchicine (blue).

Altogether, these results show that most P-gp substrates, probes and modulators interacted with the three DBSs (Fig. 3) suggesting that the DBP of the human WT P-gp model discriminate ligands differently than the internal cavity of the refined murine P-gp structure.

### Identification of Drug-Binding Sites in the human P-gp variants

To assess if the TMHs repacking found in the P-gp variants affect drug binding, docking studies using known P-gp substrates and probes were performed as previously described and compared to the WT model. The molecules top-ranked binding energies (Δ*G*) at each DBS are depicted in Table S9 of the Supporting Information.

The total repacking of the transmembrane α-helices (TMHs) observed in both substrate binding site (SBS) variants (G185V and G830V) led to changes on substrate binding mostly affecting the R and H sites. Although more dramatic in the G830V variant, most of the compounds tested that showed to interact with the three DBSs in the WT model, did not bound at the H-site or did not interact with both SBSs. Additionally, and in contrast with what was observed for the G185V variant, some of the substrates that docked at the three DBSs in WT, did not interact with both M and H sites in the G830V variant. Although, no clear conclusions could be achieved about the possible changes in the molecule’s affinity upon the TMHs rearrangement observed in these variants, nonetheless it becomes clear that the total helical repacking induced by the glycine mutations had a dramatic influence on the availability of each drug-binding site (DBS) to the evaluated set of molecules.

On the other hand, although the modulator binding site (M-site) variants (F978A and ΔF335) also showed changes on substrate binding mostly affecting the H or both SBSs, these mutations did not have a severe impact on drug binding as observed in the glycine variants. Nevertheless, due to the standard error reported for VINA and although no clear correlation could be obtained about the possible changes in drug affinity upon the TMHs repacking observed in these variants, it is quite interesting to note that specific mutations at the M-site have such a large influence on the SBSs rather than what would be initially expected, at the M-site.

Altogether, the analysis of the docking results demonstrates that all P-gp variants present changes on drug binding as a result of the total or partial TMHs repacking induced by these mutations, mostly affecting the R and H sites.

### Characterization of Drug-Binding sites

To better understand the changes on substrate binding observed in the P-gp variants, the pocket volume, residues distribution and mean polarity of each DBS were assessed for the human WT model and variants using the EPOS^BP^ software. The results were further compared to the human WT model and the refined murine P-gp structure.

Overall, both M and R sites showed similar pocket volumes among the human WT model (M-site, 1284 Å^3^ and R-site, 1902 Å^3^) and the refined murine P-gp structure (M-site, 1300 Å^3^ and R-site, 1900 Å^3^). In contrast, the H-site is considerable smaller (1232 Å^3^) in human WT than in the refined murine structure (2200 Å^3^). Additionally, and much like the refined murine structure, the M-site of the human WT showed to be the most hydrophobic DBS with higher number of aromatic residues in contrast with the H and R sites, that presented a higher percentage of polar residues. When comparing the site’s residues distribution between species, no significant changes were found in the M and R sites, although the human WT H-site is more hydrophilic than the previously reported for the murine P-gp structure. However, despite the differences in volume and residues distribution found in the human WT H-site, this SBS was capable of binding more compounds than those observed in the refined murine P-gp structure.

Regarding P-gp variants, the total repacking induced by both glycine mutations led to changes in the residues’ side-chain facing the H-site, with a remarkable increase in the content of hydrophobic (G185V, +53%; G830V, +75%) and aromatic side-chains (G830V, +63%) in respect to WT. Nevertheless, as a result of the stronger impact of G830V mutation on TMHs repacking, dramatic changes in the DBSs volume were also found. While a reduction around 50% was observed in the volume of R-site, the volumes of both M and H sites increased about 37% and 58%, respectively, in respect to WT. Therefore, for these mutations the data suggest that a slight increase in the hydrophilicity of the substrates may hamper its binding to the more hydrophobic sites.

Although the F978A mutation preferentially affected the TMHs repacking of the C-terminal P-gp halve, alterations in the residues’ side-chain facing the H-site were also observed with significant variations in the percentage of hydrophobic (+89%) and aromatic side-chains (+26%). When compared to the other P-gp variants, the ΔF335 variant seemed to show minor variations in the volume and residues’ side-chain facing all DBSs, although the H-site remained the most affected showing a decrease of its volume around 20% and an increase in the percentage of hydrophobic (+12%) and aromatic (+20%) side-chains. Similar to the glycine variants, lowering the hydrophobicity and reducing the number or aromatic rings is expected to decrease the binding to the mutated binding sites.

Altogether, these results indicate that the changes on drug binding found in all P-gp variants are related with alterations in the DBSs properties upon the rearrangement of the TMHs that delimited each DBSs. The visual inspection of each DBS showed that all mutations also induced changes in the DBSs structure, being more severe in the H-site. While in the WT model, the pocket entrance of the H-site is completely opened to the DBP with the formation of a cleft capable of accommodating substrates, all mutations induced distortions that affected either the pocket entrance and/or the cleft, compromising the binding of substrates (Fig. S23).

In sum, the characterization of the DBSs indicates that the total or partial TMHs repacking in response to mutations in the transmembrane domains (TMDs) seems to affect the shape, volume and residues distribution of the three DBSs within the drug-binding pocket (DBP). Interestingly, although changes on drug binding were also observed in the M and R sites, the H-site was the most affected by all mutations becoming unable to interact with most of the P-gp substrates and probes tested.

## Final Discussion

Modulating drug efflux by P-gp pump is one of the promising strategies to reverse MDR in cancer cells. Nevertheless, the lack of information about the molecular basis underlying drug specificity and efflux-related signal transmission mechanism between the TMD-NBD domains impairs the development of more potent and selective compounds able to overcome MDR. Therefore, to provide additional insights on drug specificity and efflux mechanism, the impact of four P-gp mutations (G185V, G830V, F978A and ΔF335), experimentally linked with changes in efflux, basal and drug-stimulated ATPase activity, were comprehensively assessed.

In this work, a human P-gp homology model was developed based on murine P-gp crystallographic structure in the *apo* state inward-facing conformation (PDB ID: 4Q9H). The initial homology model obtained was refined through MD simulations in the presence of a “linker” retrieved from a previously equilibrated murine P-gp^39^ structure, inserted in a POPC membrane also used in previous studies^122^ and thereafter validated. By comparison with a recently published cryo-EM P-gp structure^99^, our homology model was found to maintain an adequate reliability and robustness crucial for the herein proposed analysis.

The final human P-gp model was then used to thoroughly characterize the effect of the P-gp mutations mentioned above in the structure of the transporter. The mutations are located at the transmembrane region surrounding the M (F978A and ΔF335), H (G185V) and R (G830V) sites. Taken together, these mutations seem to induce TMHs repacking affecting the DBP portals by changing the “crossing helices” 4/5 and 10/11 important to NBD dimerization, and also compromising the access of drugs to the internal cavity, and by reducing the DBP volume. Additionally, as a result of the TMHs repacking significant changes in the volume, shape and polarity of the DBSs within the DBP were also observed in all P-gp variants, mostly affecting the binding of substrates at the H and R sites. It is noteworthy that although F978A and ΔF335 mutations lie at the M-site, they affect in a similar manner the SBSs properties and drug binding as observed in both glycine variants, suggesting a communication pathway between the M-site and the SBSs through the functional TMH6 and TMH12, in agreement with some experimental studies^20,22,123^. Therefore, we hypothesize that changes in the structure and polarity of the DBSs induced by the TMHs repacking i) provide a possible explanation for P-gp promiscuity, reported in the literature^13^ and ii) suggest that small variations in both substrates and modulators may be enough to impair substrate binding and/or to enhance the modulators’ activities. Interestingly, several examples on the latter are already described in literature as suitable approaches in enabling molecules to evade efflux^124^ or even to switch the activity of known substrates into high-affinity compounds able to inhibit P-gp’s ATPase activity^125^.

Even though the mutations described above directly affected the transmembrane region, they also induced changes in the total number of contacts at the ICH-NBD interfaces, suggesting that the TMHs rearrangement is involved in the TMD-NBD communication, in agreement with several experimental^10,126,127^ and *in silico* studies^8,9,45,46^. Furthermore, all P-gp variants showed significant changes in the mean contact frequencies of specific residue pairs, mainly located at the ICH2/ICH3-NBD2, an important transmission interface to couple drug binding to ATPase activity, but also being critical for P-gp folding^9,45,127,128^. Thus, another interesting approach to the modification of substrates/inhibitors is the development of allosteric modulators able to specifically interact at the ICH-NBD interfaces and impair the signal transmission between the TMD and NBDs^116^. Currently, only two scaffolds are currently known to interact in such domains, namely dihydropyridines (TMD-NBD1)^128^ and flavonoids (NBD2)^129^, and additional efforts must be taken in the future to explore this hypothesis.

Nevertheless, although all P-gp mutations induced similar structural effects on the transporters’ architecture, it seems clear that mutations at the M-site (F978A and ∆F335) have a completely different impact on P-gp structure than the mutations located at the SBSs (G185V and G830V). Both glycine mutations induced a total TMHs repacking affecting drug binding at the SBSs. Additionally, the glycine mutations do not have a significant impact in the total number of contacts as expected, suggesting that G185 (TMH3) and G830V (TMH9) residues have equivalent roles in P-gp function and possibly more involved in drug binding.

On the other hand, the effects of the M-site mutations show to be dependent of the affected TMH. The F978A mutation (TMH12) preferentially induced a partial TMH repacking, affecting drug binding at the SBSs while ΔF335 mutation (TMH6) dramatically change the residue interactions at the ICH-NBD interfaces. These results support the hypothesis that while the F978 residue is likely involved in drug binding as reported in some experimental studies^53,55,130,131^, F335 residue is involved in TMD-NBD communication^59,60^.

## Conclusion

Overall, this work provides clear evidence that mutations at these specific TMHs (i) are responsible for inducing a repacking of the TMHs, changing the DBP volume and drug binding sites, mostly affecting drug binding at the SBSs and (ii) also impact the ICH-NBD signal transmission interfaces, suggesting that a perturbation in the TMDs (e.g. mutations or binding of substrates) induce a TMHs rearrangement that are transmitted to the NBDs through changes in the residue interactions between the ICHs and the respective NBD, in agreement with experimental studies^61,130,131^.

Nevertheless, as protein conformational changes may occur at least on the timescale of microseconds^132^ and the computational power available is limited, it should be clear that our findings are based in theoretical models aiming to predict the possible structural impact of single-point mutations on P-gp architecture/organization. Additionally, as these P-gp mutations are experimentally related with changes in drug-stimulated ATPase activity upon binding of specific substrates, more studies are needed to assess their direct impact on drug and ATP binding. Therefore, further studies including molecules with altered efflux properties are undergoing, in an attempt to clarify these issues.

## Supplementary information

Supplementary Information.

Supplementary Information2.

## Data Availability

The final configurations of the MD refined human P-gp homology structure and its variants (G185V, G830V, F978A and ΔF335) are available for download at our website (http://chemistrybits.com/).
