# Supplementary material for: Theoretical insights on helix repacking as the origin of P-glycoprotein promiscuity
Source: Sci Rep. 2020 Jun 17;10:9823. doi: 10.1038/s41598-020-66587-5 (PMC7300024; doi:10.1038/s41598-020-66587-5)
Supplement: Supplementary file 1 — Supplementary Information. [file 41598_2020_66587_MOESM1_ESM.pdf]

SUPPORTING INFORMATION

"Theoretical insights on helix repacking as the origin of P-glycoprotein promiscuity"

Cátia A. Bonito,[1] Ricardo J. Ferreira,[2] Maria-José. U. Ferreira,[3] Jean-Pierre Gillet,[4] M. Natália D. S. Cordeiro[1], Daniel J. V. A. dos Santos[1,5]*

[1] LAQV@REQUIMTE, Department of Chemistry and Biochemistry, Faculty of Sciences, University of Porto, Rua do Campo Alegre, 4169-007 Porto, Portugal; [2] Department of Cell and Molecular biology- Molecular Biophysics, Uppsala University, Husargatan 3, Sweden; [3] Research Institute for Medicines (iMed.ULisboa), Faculty of Pharmacy, Universidade de Lisboa, Av. Prof. Gama Pinto, 1649-003 Lisboa, Portugal; [4] Laboratory of Molecular Cancer Biology, Molecular Physiology Research Unit-URPhyM, Namur Research Institute for Life Sciences (NARILIS), Faculty of Medicine, University of Namur, B-5000 Namur, Belgium; [5] BioISI: Biosystems and Integrative Sciences Institute, Faculty of Sciences, University of Lisbon Campo Grande, C8, 1749–016, Lisbon, Portugal

Table of Contents

Figure S1. Schematic representation of the fully unrestrained *NpT* runs performed to obtain a stable human P-gp homology model and variants.
Figure S2. Ramachandran plots of initial and final homology models.
Figure S3. Root mean square deviation (RMSD) of the human P-gp v3 models during MD simulations.
Table S1. Structural quality assessment of the human P-pg homology models.
Figure S4. Evaluation of membrane insertion energies for the *in-house* P-gp model and cryo-EM human P-gp model (PDB ID: 6QEX) obtained from QMEMBrane server.
Figures S5-S6. Superimposition of the initial template (4Q9H), the homology model (v4c) and human P-gp cryo-EM structure (6QEX).
Table S2. RMSD values for each TMH between the human WT P-gp model (v3c) and 6QEX human P-gp structure. The RMSD values of the TMHs 4 and 10 between the WT model and human/mouse P-gp structures.
Figure S7. Location of the selected mutations in the human P-gp model and root mean square fluctuations (RMSF) comparison for all models (WT, G185V, G830V, F978A and ΔF335).
Figure S8. Probability distribution function P(V) and cumulative distribution of the DBP volumes in the human WT P-gp model and variants.
Tables S3-S8 and **Figures S9-S20**. Results from *g_bundle* analysis.
Table S9. Top-ranked binding energies (Δ*G*) in kcal.mol-1 obtained for each site within the DBP of the human WT P-gp model and variants.
Figure S21. Comparison of total number of contacts at each ICH-NBD interface for the WT model and all P-gp variants (obtained with *g_hbond*).
Figure S22. Variation in the total number of contacts found at each ICH-NBD interface, for all P-gp variants, when compared to the WT.
Table S10. Contact frequencies between ICH and NBD residues for all coupling helices.
Figure S23. Graphical representation of the molecular surface for the H-site in the human WT P-gp model and variants.
Figure S24. Docking results for taxol molecule in the 6QEX and homology P-gp models.

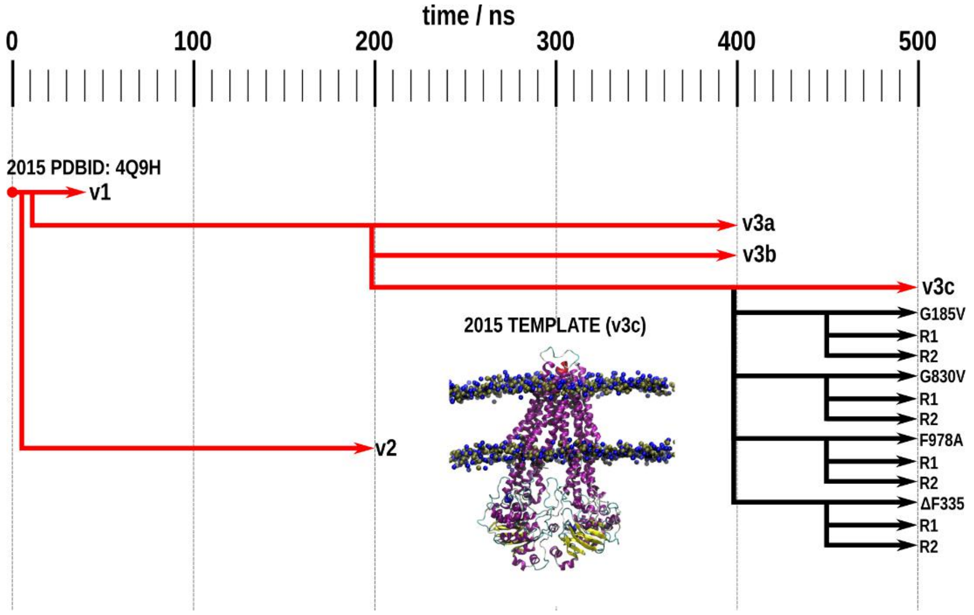

Figure S1. Schematic representation of the fully unrestrained *NpT* runs performed to obtain a stable human P-gp homology model and variants. The murine template used are colored as red (PDB ID:4Q9H), and the mutated structures in black.

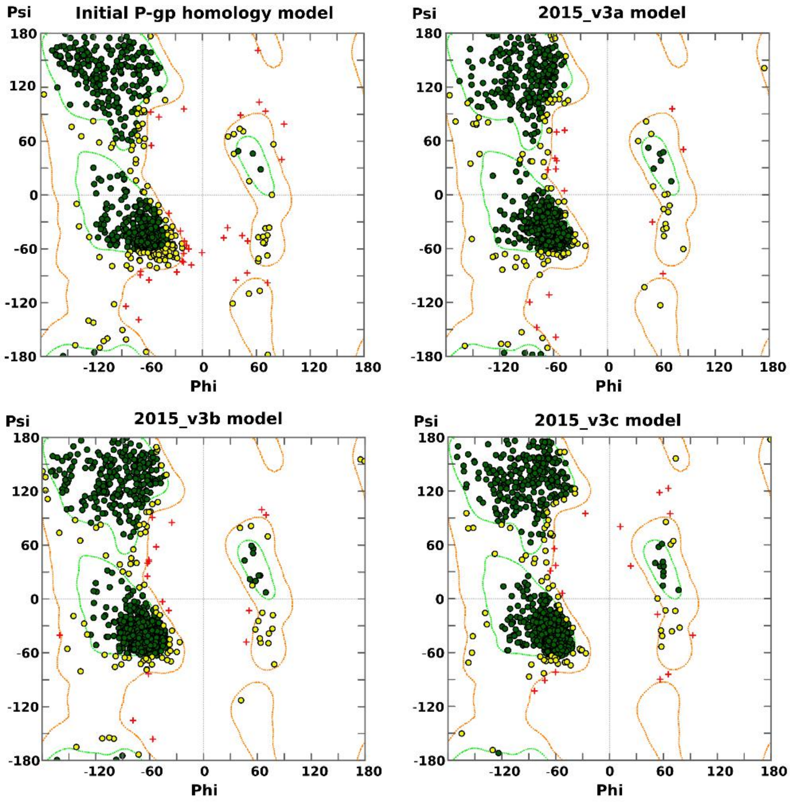

Figure S2. Ramachandran plots of the initial and refined human P-gp models after 400 ns MD simulations. The outliers are represented as red crosses, allowed positions in yellow and core angles in green.

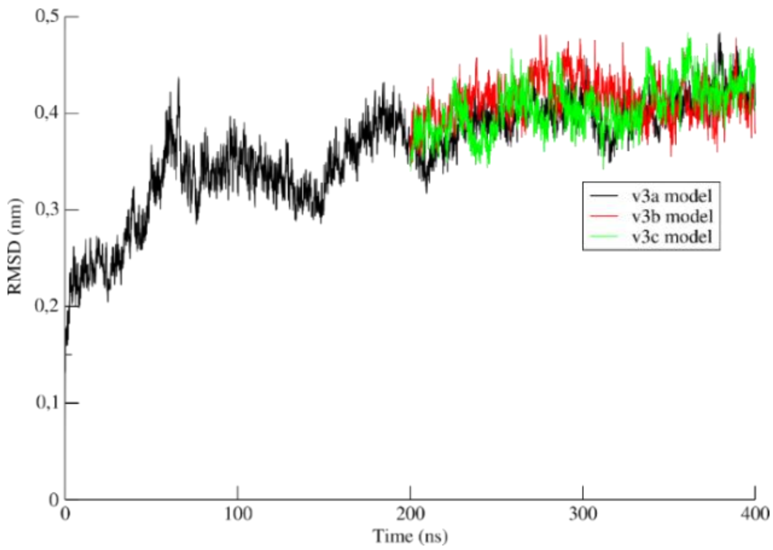

Figure S3. Root mean square deviation (RMSD) of the human P-gp v3 models during MD simulations.

Table S1. Structural quality assessment of the human P-pg homology models

		Assessment software						
		ERRAT	MOLPROBITY		SwissModel		PROCHEK	
			Score	Percentile	QMEAN6	Z-Score	Morris	G-factors
**Crystal (PDB ID: 4Q9H)**	w/o linker	90.05	1.86	83	0.61	-1.75	1-2-2	0.18
**Homology model**	w/o linker	74.40	2.22	63	0.59	-1.89	1-2-3	-0.41
	w/ linker	75.08	2.21	64	0.58	-2.05	1-2-3	-0.41
**MD Refined P-gp models**	V3a	93.42	1.71	89	0.46	-3.39	1-2-2	-0.61
	V3b	96.65	1.64	91	0.44	-3.52	1-2-2	-0.58
	V3c	97.79	1.65	91	0.43	-3.68	1-2-2	-0.57
**Cryo-EM (PDB ID: 6QEX)**	w/o linker	85.54	1.78	86	0.70	-1.65	1-2-2	0.06

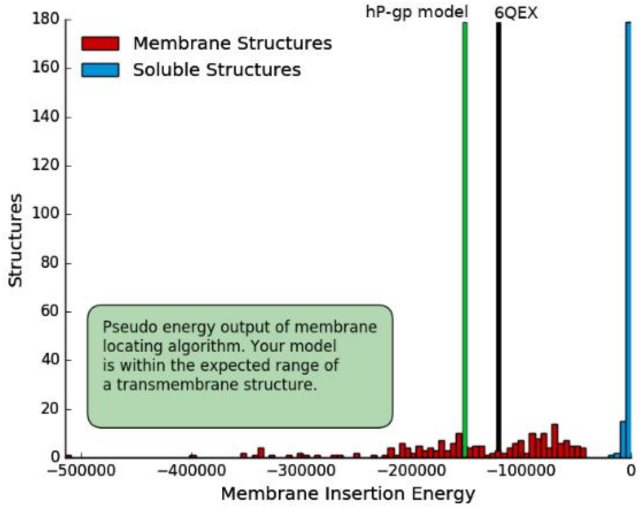

Figure S4. Evaluation of membrane insertion energies for the *in-house* P-gp model and cryo-EM human P-gp model (PDB ID: 6QEX) obtained from QMEMBrane server.

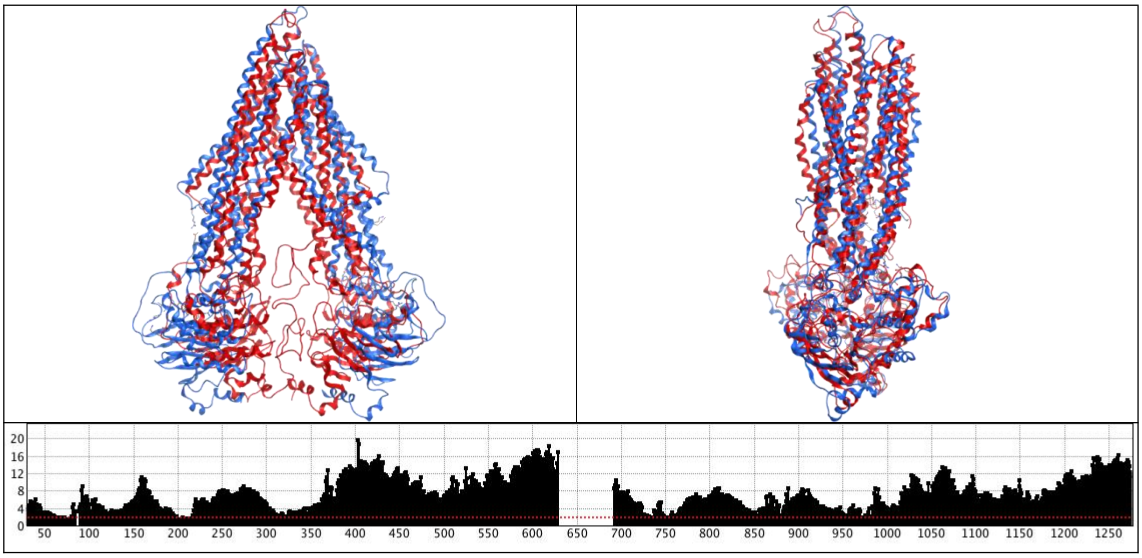

Figure S5. Root-mean square deviation (total RMSD: 8.003) between the template (4Q9H, blue) and the final homology model (v3c, red) structures (top) and residue-by-residue (bottom). RMSD values are depicted in angstroms.

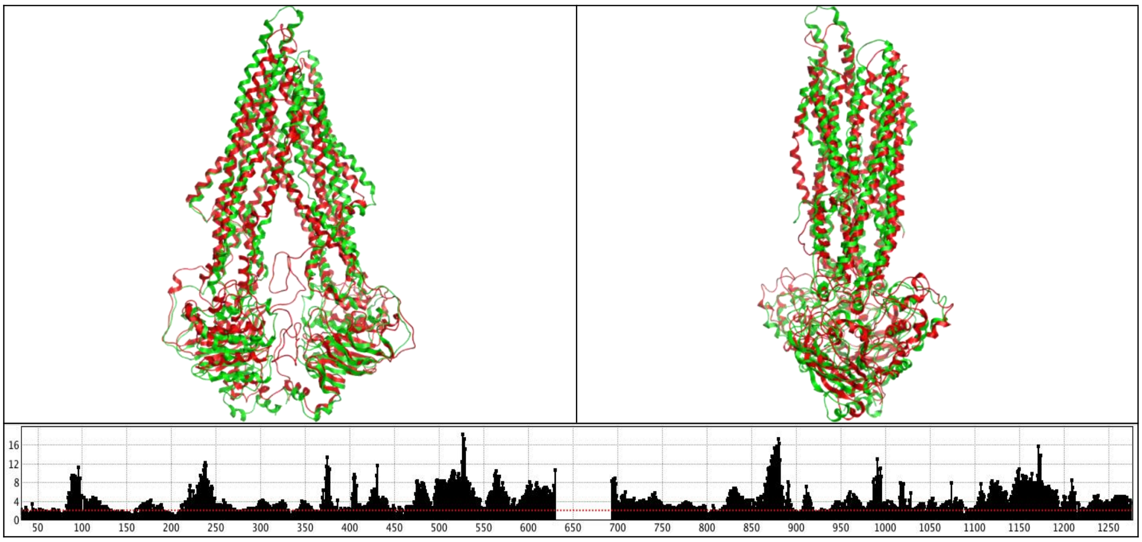

Figure S6. Root-mean square deviation (total RMSD = 4.931) between the cryo-EM (6QEX, green) and the final homology model (v3c, red) structures (top) and residue-by-residue (bottom). RMSD values are depicted in angstroms.

Table S2. RMSD values for each TMH between the human WT P-gp model (v3c) and 6QEX human P-gp structure. The RMSD values of the TMHs 4 and 10 between the WT model and human/mouse P-gp structures.

**PDB**	TM1	TM2	TM3	TM4	TM5	TM6	TM7	TM8	TM9	TM10	TM11	TM12
6QEX	1.71	1.57	1.59	6.07	1.13	2.08	1.43	1.62	1.61	4.94	1.73	3.34
6FN4				4.87						4.89		
6GDI				1.07						2.86		
5KPI				1.51						2.61		
4Q9H				1.58						2.72		
4M1M				1.42						2.72		
4M2S				1.42						2.66		
4Q9L				1.64						2.76		

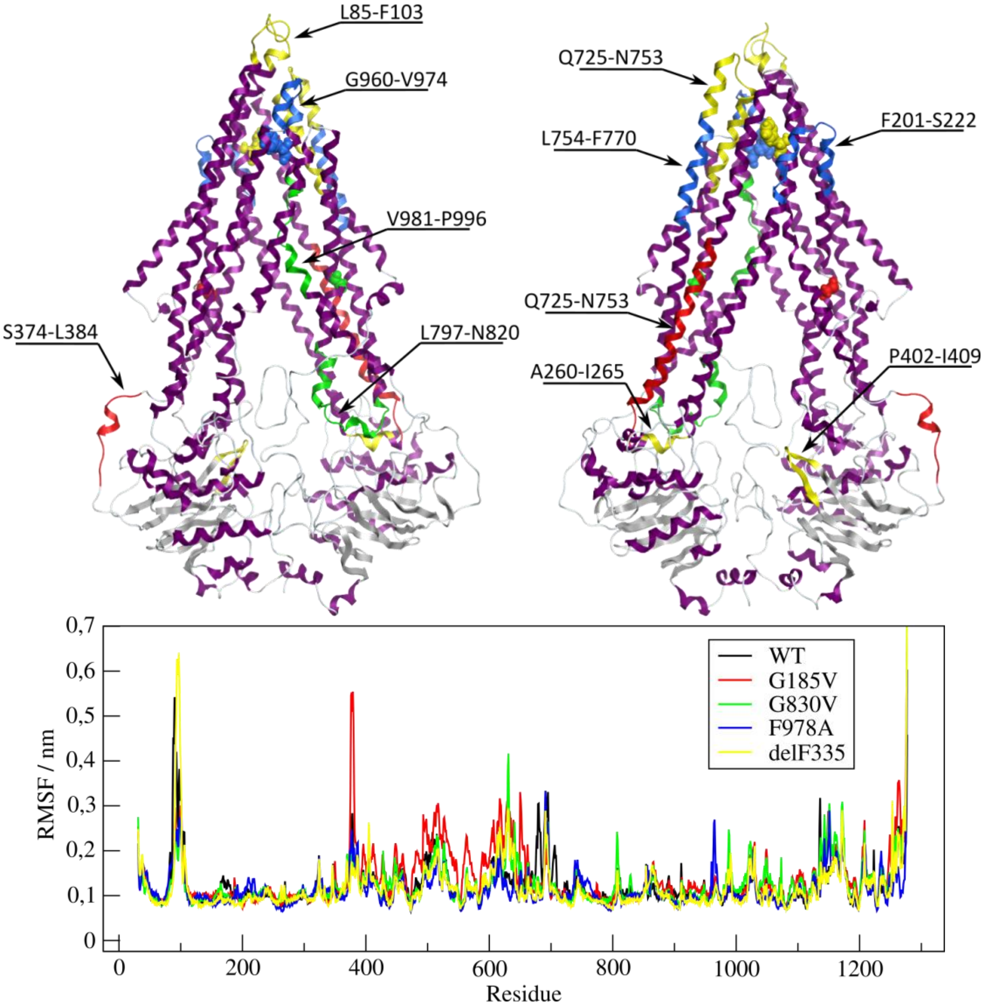

Figure S7. (top) Location of the selected mutations (in van der Waals, G185V, red; G830V, green; F978A, blue; ΔF335, yellow) in the human WT P-gp model. Aminoacid sequences with larger RMSF differences from WT model are depicted with the same color code; (bottom), comparison of root-mean square fluctuations (RMSF) from mutated P-gp models against WT (black).

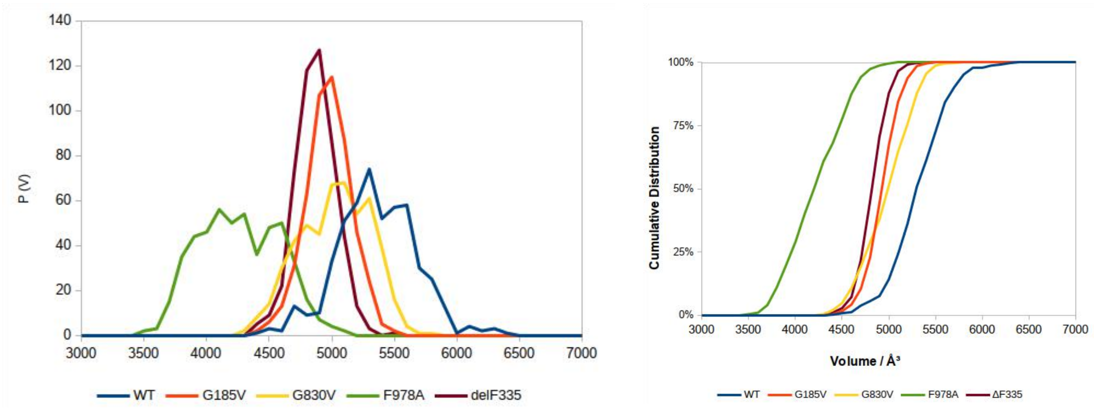

Figure S8. Probability distribution function P(V) and cumulative distribution of the DBP volumes in the human WT P-gp model and variants.

Table S3. Variation in the axis distance (*bun_dist*) observed in the P-gp variants transmembrane helices (compared to the WT; +, positive mean changes; –, negative changes).

	TM1	TM2	TM3	TM4	TM5	TM6	TM7	TM8	TM9	TM10	TM11	TM12
**G185V**						+			–			
**G830V**								–	–			
**F978A**									–			
**ΔF335**									–			

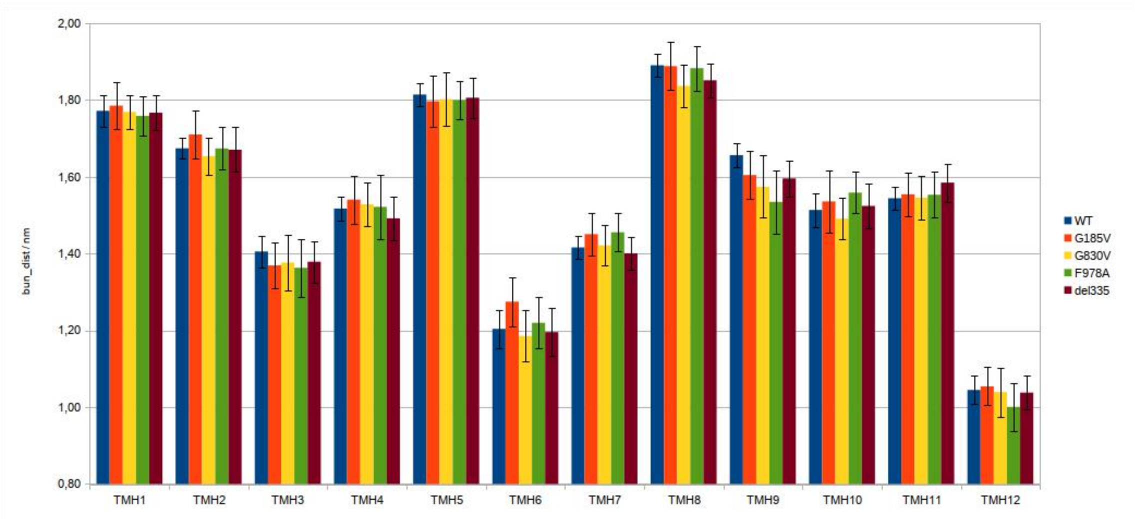

Figure S9. Comparison of individual transmembrane axis distance from the bundle center for all P-gp variants.

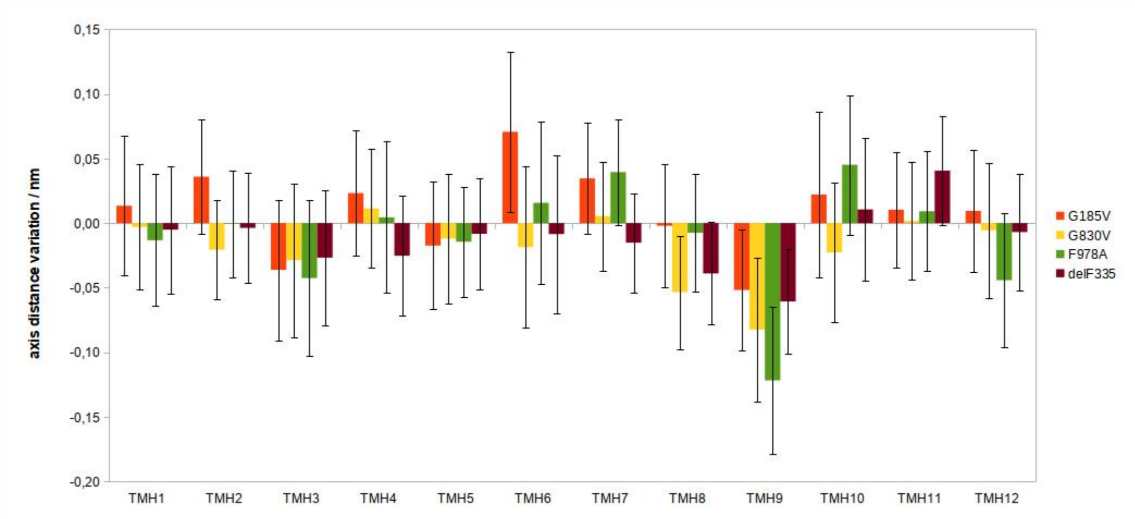

Figure S10. Variation in the transmembrane axis distance from the bundle center for all P-gp variants (difference from WT).

Table S4. Variation in the transmembrane axis length (*bun_len*) observed in all P-gp variants (compared to the WT; +, positive mean changes; –, negative changes).

	TM1	TM2	TM3	TM4	TM5	TM6	TM7	TM8	TM9	TM10	TM11	TM12
G185V										–	–	–
G830V									+	–		+
F978A						–	+				–	
ΔF335											–	–

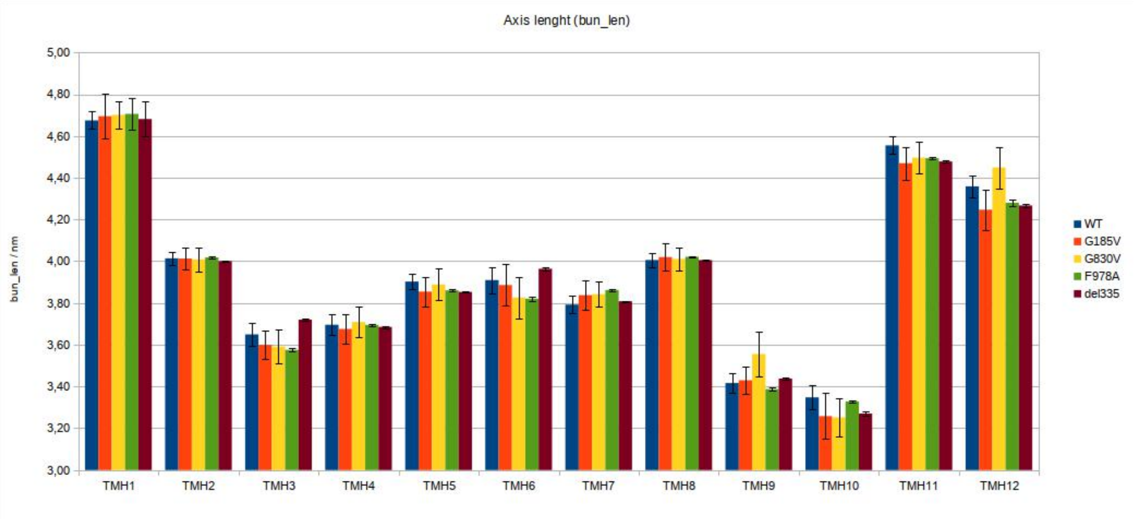

Figure S11. Comparison of individual transmembrane axis length for all P-gp variants.

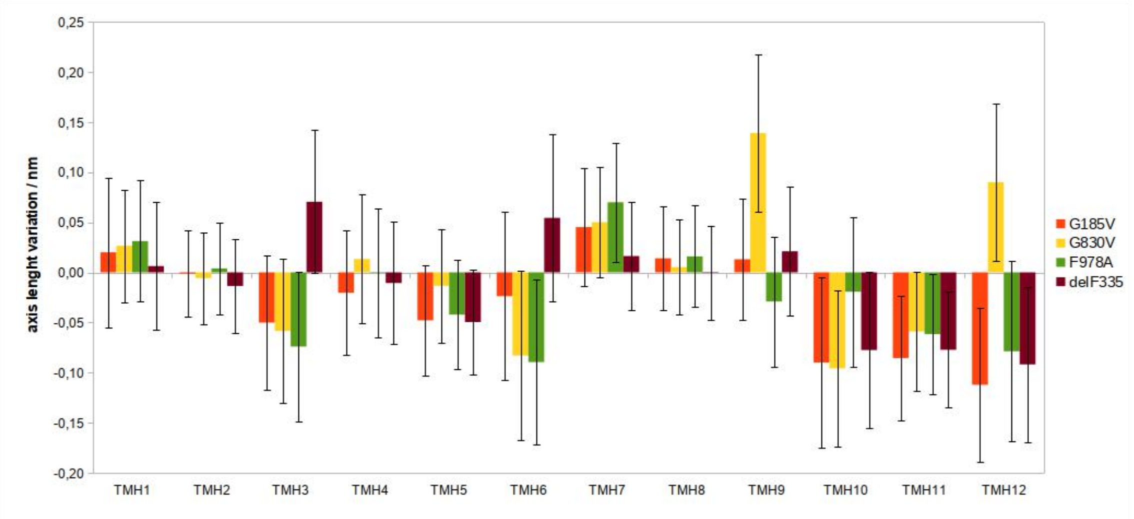

Figure S12. Variation in the transmembrane axis length for all P-gp variants (difference from WT).

Table S5. Variation in the *z*-shift of the axis mid-points (*bun_z*) observed in the P-gp variants transmembrane helices (compared to the WT; +, positive mean changes; –, negative changes).

	TM1	TM2	TM3	TM4	TM5	TM6	TM7	TM8	TM9	TM10	TM11	TM12
G185V							+			+		
G830V			–	–	–	–	+			+		+
F978A				–			+		–	+		–
ΔF335											–	

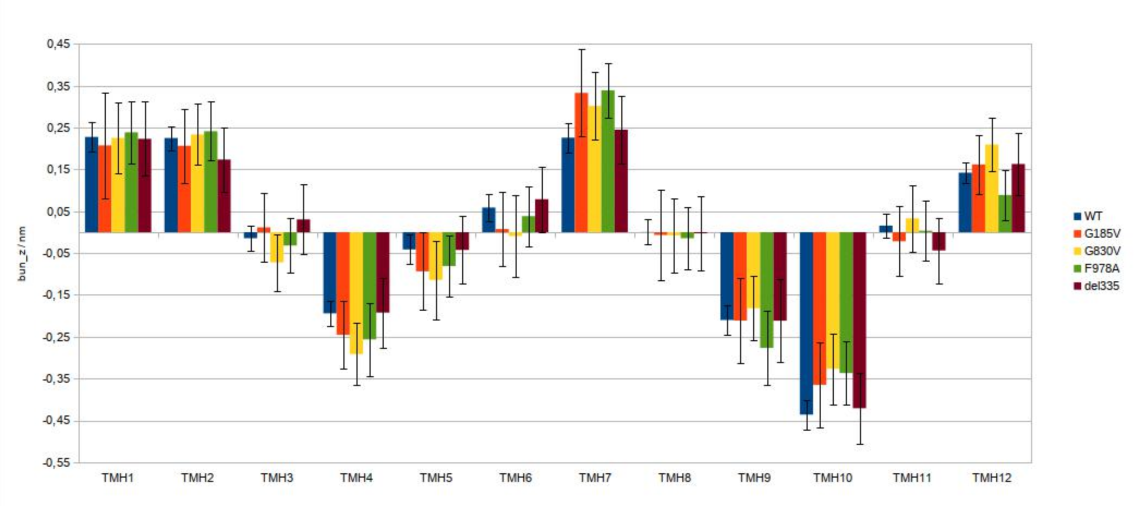

Figure S13. Comparison of individual transmembrane *z*-shifts, from the axis mid-points, for all P-gp variants.

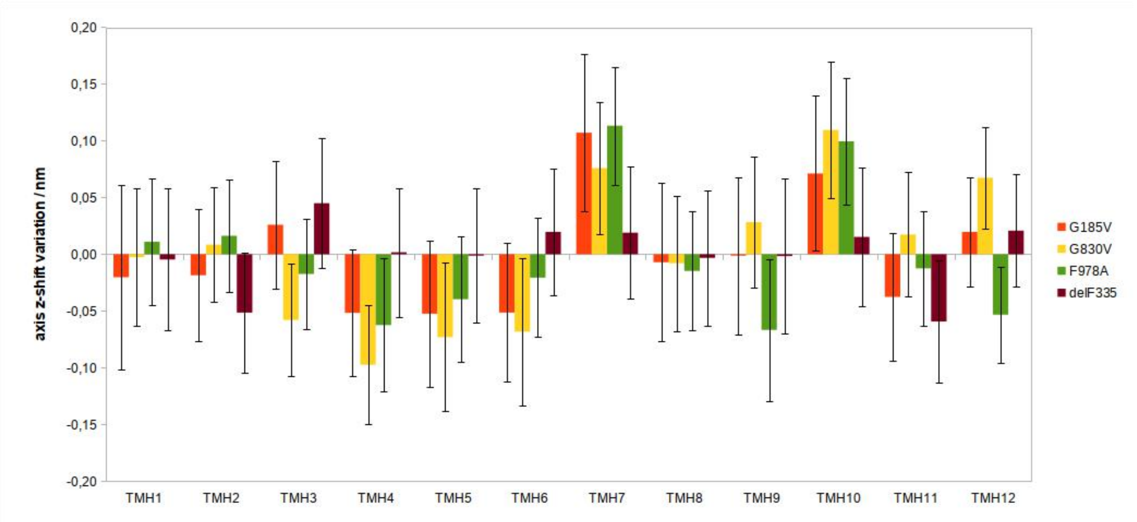

Figure S14. Variation in the transmembrane *z*-shift of the axis mid-points for all P-gp variants (difference from WT).

Table S6. Variation in the axis total tilt (*bun_tilt*) observed in the P-gp variants transmembrane helices (compared to the WT; +, positive mean changes; –, negative changes).

	TM1	TM2	TM3	TM4	TM5	TM6	TM7	TM8	TM9	TM10	TM11	TM12
G185V			+					+				
G830V		–	+		–			+			–	+
F978A								+				
ΔF335												

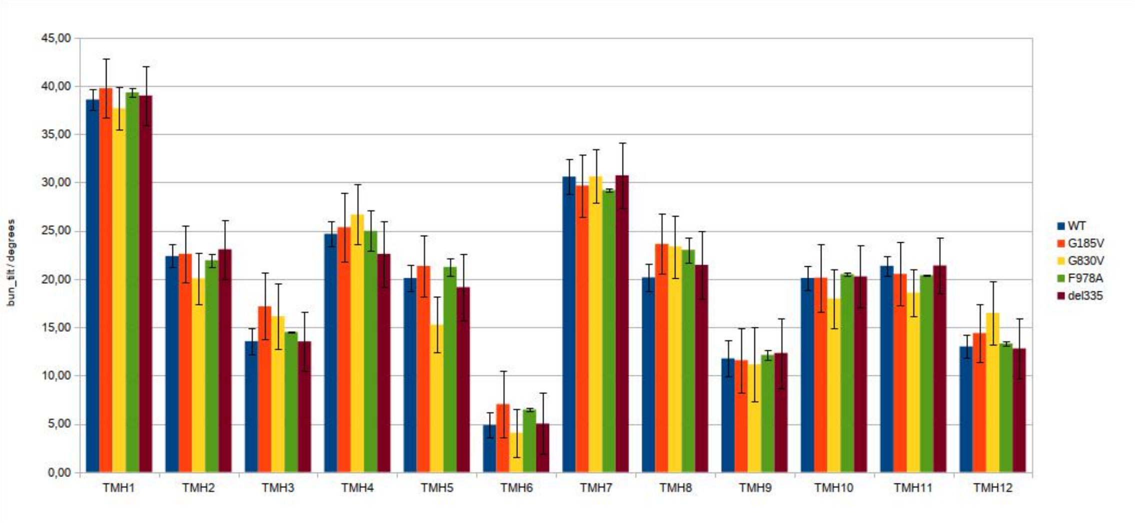

Figure S15. Comparison of individual transmembrane axis total tilt, against *z* axis, for all P-gp variants.

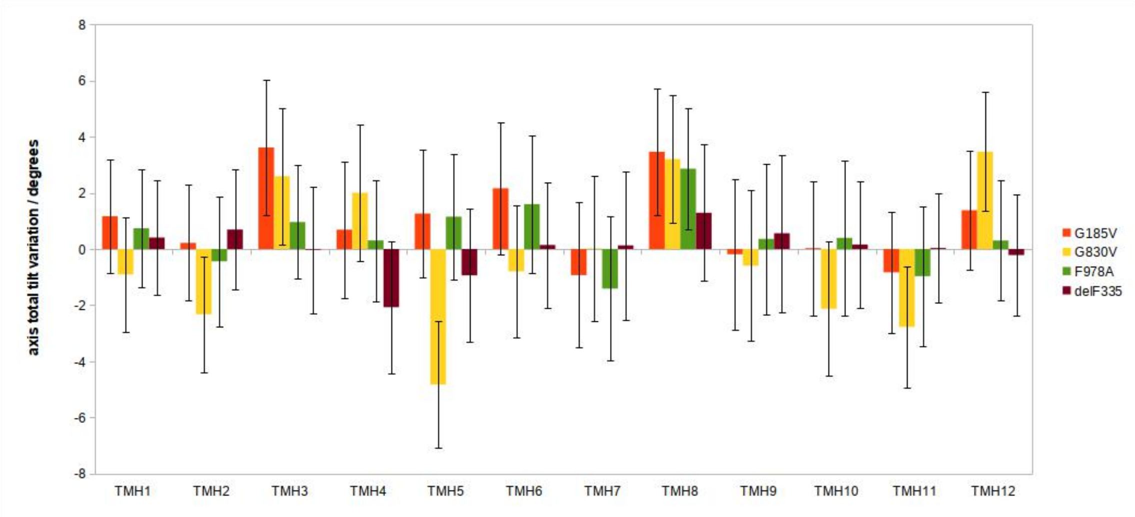

Figure S16. Variation in the transmembrane axis total tilt, against *z* axis, for all P-gp variants (difference from WT).

Table S7. Variation in the axis lateral tilt (*bun_tiltl*) observed in the P-gp variants transmembrane helices (compared to the WT; +, positive mean changes; –, negative changes).

	TM1	TM2	TM3	TM4	TM5	TM6	TM7	TM8	TM9	TM10	TM11	TM12
G185V	+		+			+		–				
G830V	+	+			–		–	–			+	
F978A	+					+	–	–				
ΔF335				+		+	+					

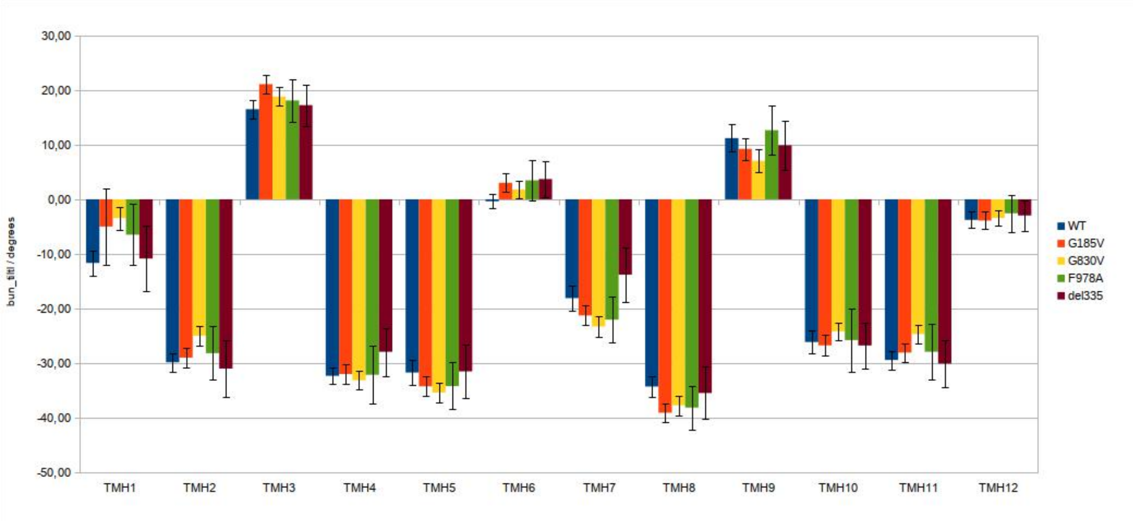

Figure S17. Comparison of individual transmembrane axis lateral tilt, against *z* axis, for all P-gp variants.

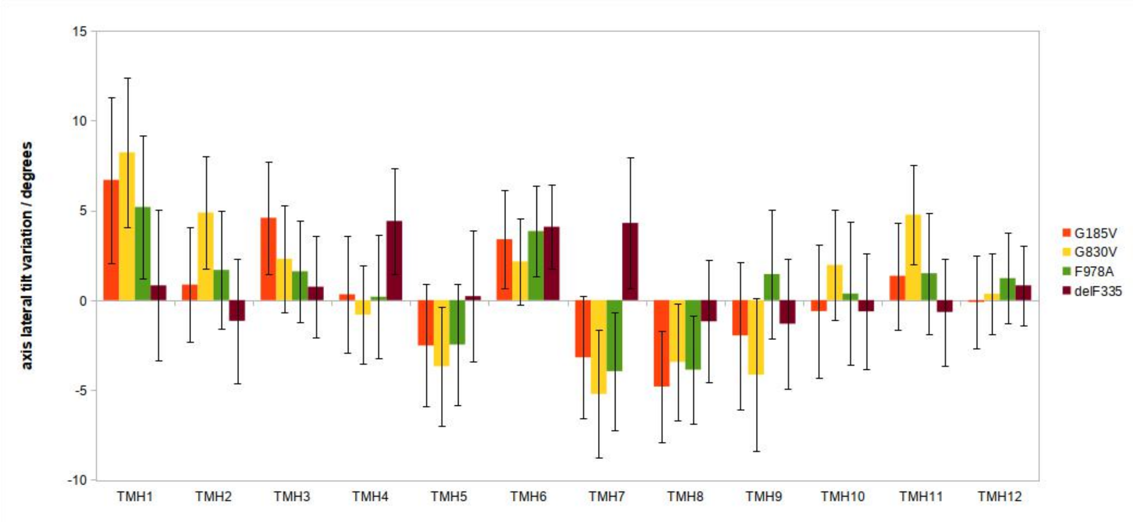

Figure S18. Variation in the transmembrane axis lateral tilt, against *z* axis, for all P-gp variants (difference from WT).

Table S8. Variation in the axis radial tilt (*bun_tiltr*) observed in the P-gp variants transmembrane helices (compared to the WT; +, positive mean changes; –, negative changes).

	TM1	TM2	TM3	TM4	TM5	TM6	TM7	TM8	TM9	TM10	TM11	TM12
G185V					+							
G830V				+	+	+				–		
F978A					+		+			+		
ΔF335					+							

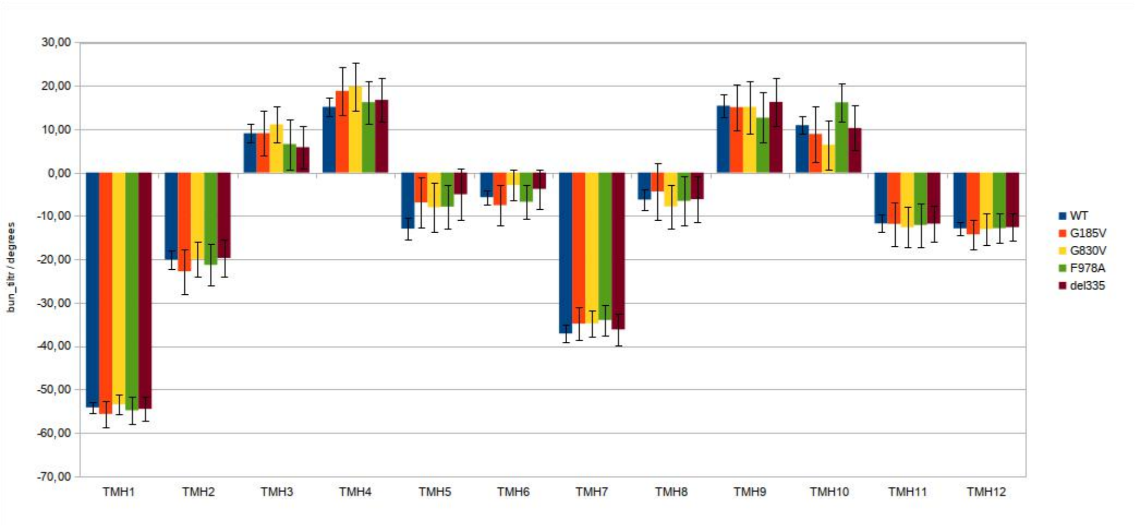

Figure S19. Comparison of individual transmembrane axis lateral tilt, against the bundle axis, for all P-gp variants.

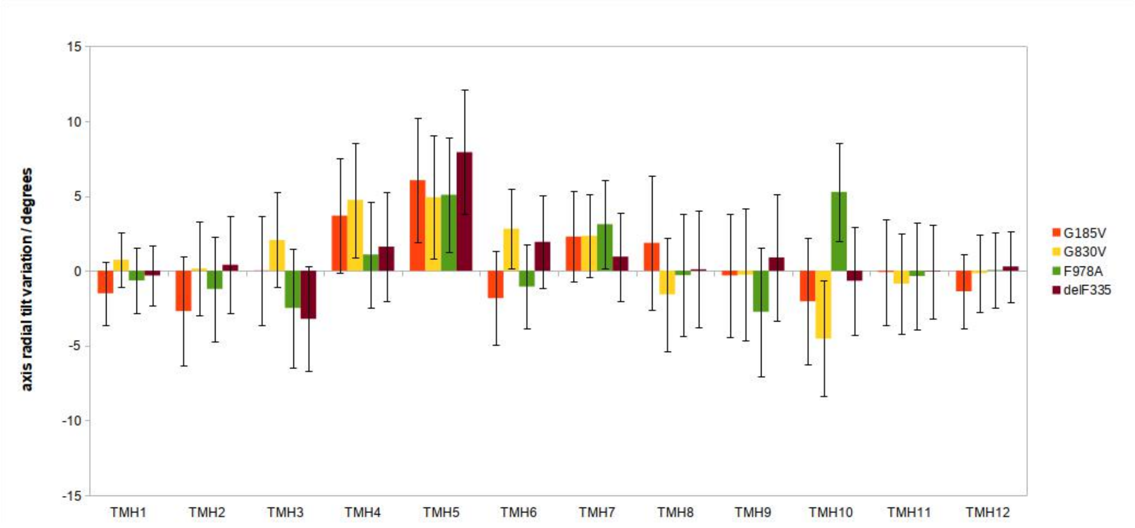

Figure S20. Variation in the transmembrane axis radial tilt, against the bundle axis, for all P-gp variants (difference from WT).

Identification of Drug-Binding Sites in human P-gp variants

Table S9. Top-ranked binding energies (Δ*G*) in kcal.mol^{-1} obtained for each site within the DBP of the human WT P-gp model and variants.

	P-gp WT (ΔG; kcal.mol-1)			G185V (ΔG; kcal.mol-1)			G830V (ΔG; kcal.mol-1)			F978A (ΔG; kcal.mol-1)			ΔF335 (ΔG; kcal.mol-1)		
**SUBSTRATES**	**M-site**	**R-site**	**H-site**	**M-site**	**R-site**	**H-site**	**M-site**	**R-site**	**H-site**	**M-site**	**R-site**	**H-site**	**M-site**	**R-site**	**H-site**
Actinomycin D		-11,2			-11,5			-11,3			-12,4			-11,7	
Amprenavir	-8,6			-8,5			-9,0			-8,2			-8,2		
Bromocriptine	-10,0	-8,9	-8,4	-10,4	-9,5		-11,0			-10,0	-9,6		-10,5	-8,9	-8,9
Calcein-AM	-7,9	-7,8		-8,2	-8,4		-8,7			-8,0	-8,0		-7,7		
Calcein	-6,9	-7,7	-7,1	-7,6	-7,2		-9,4			-7,5	-7,3		-7,7	-7,9	
Chloroquine	-7,2	-6,8		-7,2			-7,7			-6,8			-7,3		
Colchicine	-8,7	-7,4	-6,8	-10,8	-7,0		-8,7	-7,8		-7,7			-8,1	-6,8	
Cyclosporine		-7,9			-9,0			-9,1			-9,6			-8,9	
Daunorubicin	-8,5	-8,3	-8,2	-9,1	-8,8		-8,0	-8,2		-9,2			-9,2	-7,9	
Dexamethasone	-8,5	-8,1	-8,2	-9,3	-8,4			-9,2		-8,9			-8,7		-7,5
Digoxigenin	-8,3	-8,0	-9,0	-8,4	-7,9			-10,3		-9,4			-8,8	-7,8	-7,7
Monotoxoside	-9,8	-8,9	-8,6	-10,1	-9,5			-10,6		-10,3	-9,2		-9,8	-8,6	-8,9
Digitoxoside	-9,1	-10,4	-9,1	-10,3	-9,6			-11,3		-11,1	-10,3		-10,2	-10,0	
Digoxin	-9,9	-9,9	-9,6		-10,7			-11,0		-11,6	-12,0			-11,6	
Diphenhydramine	-7,5			-6,7				-7,3		-7,0			-6,8		-6,1
Doxorubicin	-8,4	-8,3	-8,7	-8,6	-8,0		-10,1			-9,2	-9,2	-8,0	-9,2	-8,5	
Erythromycin		-7,7	-7,9		-7,9		-7,4			-8,3	-8,4		-7,6		
Etoposide	-8,4	-8,2	-8,0	-8,9	-9,3		-9,9			-8,8			-9,0		
Hoechst 33258	-9,9	-9,4	-8,4	-9,5	-9,3			-10,4		-9,5	-10,2		-9,2	-10,9	
Hoechst 33342	-9,7	-8,6		-9,4	-9,1		-10,6	-8,7		-9,7	-9,2		-9,9	-8,4	
Indinavir	-9,4	-8,9		-10,2				-10,4		-9,2			-9,6	-9,0	
Itraconazole	-10,1	-9,5			-10,1			-10,5			-10,5			-11,1	
Ketoconazole	-9,5	-9,2		-9,5	-8,7		-10,7			-8,8	-9,2		-9,3	-9,0	
LDS-751	-7,6	-7,5		-7,9	-7,0		-8,8			-8,1	-8,1		-7,7	-7,9	-7,8
Methotrexate	-7,8	-7,7	-7,4	-8,3	-8,0		-8,9			-8,2	-8,7		-8,4	-7,9	
Midazolan	-9,1	-7,6	-7,0	-9,6			-9,1			-8,6	-7,5		-8,8		
Prazosin	-7,7	-7,6	-6,8	-8,0	-7,3		-8,8			-7,4	-7,6		-7,5	-8,1	-7,1
Progesterone	-8,5	-7,9	-8,4	-9,5			-10,3			-9,3			-9,3		
Quercetin	-7,9	-7,7	-7,2	-8,1			-7,6			-7,8	-8,1	-7,1	-7,7	-8,3	-7,5
Quinidine	-8,3	-8,1	-7,8	-8,1			-9,1			-7,9			-8,8		
R-propranolol	-7,1			-6,5			-7,8			-6,9			-7,2		-7,0
R-verapamil	-7,3	-6,9		-7,8			-8,7			-7,0			-7,5		
Reserpine	-8,7	-9,2		-9,0	-8,2		-10,2			-8,9	-8,8		-9,3	-8,6	
Rhodamine-123	-8,8		-7,1	-8,4	-7,5		-9,1				-8,1		-7,6		
Ritonavir	-8,3	-8,3	-7,7	-9,5	-8,7		-9,2			-9,1			-8,8		
S-propranolol	-6,8			-6,9			-8,1			-6,7	-6,5		-7,5	-7,2	
S-verapamil	-7,5	-6,9	-6,9	-7,7			-8,4			-7,7			-7,6		
Taxol (Paclitaxel)	-9,1	-8,9	-8,9	-9,0	-8,4		-9,8			-9,8	-9,6		-10,6		-9,8
Trimethoprim	-7,4	-6,5		-6,8			-6,9			-6,5	-6,5		-6,9	-6,3	
Vinblastine		-7,2	-6,8	-8,9	-8,6		-7,4	-7,3	-7,3		-9,0		-9,2		-8,8
Yohimbine	-8,9	-8,3	-7,8	-9,0					-9,4	-8,9	-8,2		-9,0	-8,0	-7,6

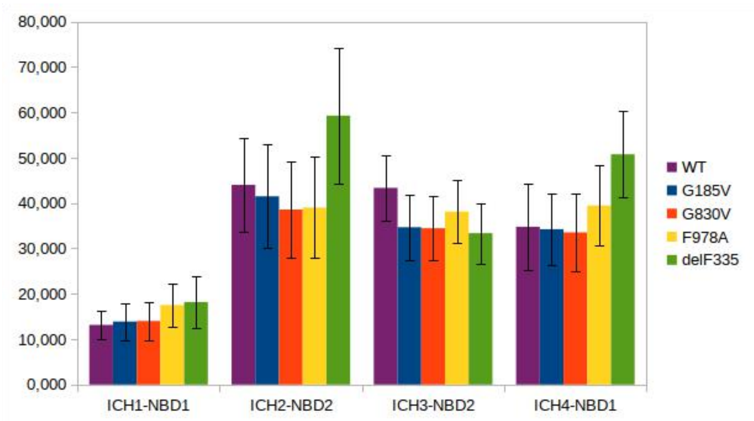

Figure S21. Comparison of total number of contacts at each ICH-NBD interface for the WT model and all P-gp variants (obtained with *gmx hbond*).

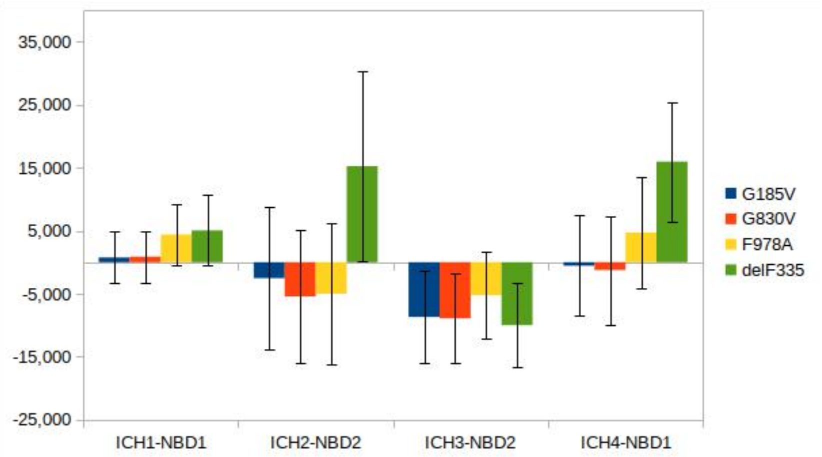

Figure S22. Variation in the total number of contacts found at each ICH-NBD interface, for all P-gp variants, when compared to the WT.

Table S10. Contact frequencies between ICH-NBD residues in the human WT P-gp model and variants.

	WT		G185V		G830V		F978A		delF335	
ICH1	NBD1	Frequency	Variation		Variation		Variation		Variation	
160 ILE	401 TYR	0,133733	0,069195	-6%	0,100466	-3%	0,065203	-7%	0,089820	-4%
**160 ILE**	**443 LEU**	0,125749	0,531604	41%	0,345309	22%	0,781770	66%	0,533600	41%
**160 ILE**	**444 TYR**	0,107784	0,188290	8%	0,157685	5%	0,159016	5%	0,125083	2%
164 ASP	403 SER	0,023952	0,033267	1%	0,041916	2%	0,067864	4%	0,228210	20%
164 ASP	404 ARG	0,990020	0,917498	-7%	0,931470	-6%	0,974717	-2%	0,956753	-3%
164 ASP	405 LYS	0,946108	0,889554	-6%	0,892881	-5%	0,717232	-23%	0,165003	-78%

Hydrogen bonds	life	1218,345	762,167	-37%	687,587	-44%	659,417	-46%	521,357	-94%
	<N>	1,960	2,109	8%	1,998	2%	2,415	23%	2,094	7%
	DG	-22,542	-21,105	-6%	-6,904	-69%	-20,994	-7%	-20,002	-11%

	WT		G185V		G830V		F978A		delF335	
ICH4	NBD1	Frequency	Variation		Variation		Variation		Variation	
904 PHE	443 LEU	0,259481	0,326680	7%	0,341317	8,18%	0,354624	10%	0,109115	-15%
**905 ARG**	**401 TYR**	0,117764	0,230872	11%	0,213573	9,58%	0,395875	28%	0,643380	53%
**905 ARG**	**434 SER**	0,255489	0,051231	-20%	0,128410	-12,71%	0,041916	-21%	0,021291	-23%
**905 ARG**	**438 GLN**	0,365269	0,151031	-21%	0,190286	-17,50%	0,358616	-1%	0,727212	36%
**905 ARG**	**441 GLN**	1,000000	0,699268	-30%	1,000000	0,00%	0,999335	0%	0,996008	0%
906 THR	441 GLN	0,159681	0,160346	0%	0,121757	-3,79%	0,149035	-1%	0,214904	6%
906 THR	474 SER	0,203593	0,179641	-2%	0,038589	-16,50%	0,384564	18%	0,136394	-7%
907 VAL	480 PHE								0,306720	new
**908 VAL**	**467 ARG**	0,548902	0,783766	23%	0,715902	16,70%	0,684631	14%	0,767132	22%
**909 SER**	**441 GLN**	0,986028	0,928144	-6%	0,931471	-5,46%	0,992016	1%	0,995343	1%
**909 SER**	**467 ARG**	0,069860	0,218230	15%	0,105788	3,59%	0,089820	2%	0,114438	4%
**909 SER**	**472 VAL**	0,151697	0,143047	-1%	0,139721	-1,20%	0,159015	1%	0,282768	13%
910 LEU	467 ARG	0,233533	0,420492	19%	0,542914	30,94%	0,000665	-23%	0,003992	-23%
910 LEU	547 ARG								0,413174	new
911 THR	467 ARG	0,293413	0,000665	-29%	0,060545	-23,29%	0,781770	49%	0,893546	60%
912 GLN	464 ARG	0,311377	0,460413	15%	0,393879	8,25%	0,352628	4%	0,321357	1%
912 GLN	467 ARG	0,231537	0,598137	37%	0,139721	-9,18%	0,195609	-4%	0,083832	-15%
913 GLU	464 ARG								0,038589	new
914 GLN	464 ARG								0,212242	new

Hydrogen bonds	life	682,765	311,115	-54%	392,277	-42,55%	689,296	1%	366,428	-46%
	<N>	2,760	2,084	-24%	2,371	-14,08%	2,805	2%	3,622	31%
	DG	-21,084	-19,012	-10%	-19,661	-6,75%	-20,876	-1%	-19,374	-8%

	WT		G185V		G830V		F978A		delF335	
ICH2	NBD2	Frequency	Frequency	Variation	Frequency	Variation	Frequency	Variation	Frequency	Variation
262 ARG	1044 TYR	0,331337	0,258150	-7%	0,223553	-11%	0,242182	-9%	0,202262	-13%
262 ARG	1077 SER	0,522954	0,456420	-7%	0,427146	-10%	0,280106	-24%	0,384564	-14%
262 ARG	1081 GLN	0,526946	0,417831	-11%	0,533599	1%	0,394544	-13%	0,519627	-1%
262 ARG	1086 PHE	0,399202	0,538257	14%	0,522954	12%	0,507651	11%	0,326015	-7%
262 ARG	1200 ASP	0,081836	0,067864	-1%	0,075848	-1%	0,039255	-4%	0,222222	14%
263 THR	1117 SER	0,642715	0,604125	-4%	0,475050	-17%	0,705922	6%	0,473054	-17%
263 THR	1118 GLN	0,023952	0,162342	14%	0,031936	1%	0,053892	3%	0,015968	-1%
263 THR	1200 ASP	0,033932	0,224218	19%	0,030605	0%	0,057884	2%	0,014637	-2%
265 ILE	1086 PHE	0,279441	0,175649	-10%	0,244178	-4%	0,206920	-7%	0,292748	1%
265 ILE	1110 ARG	0,754491	0,592149	-16%	0,477711	-28%	0,592149	-16%	0,350632	-40%
266 ALA	1086 PHE	0,570858	0,500998	-7%	0,431138	-14%	0,522954	-5%	0,383899	-19%
267 PHE	1110 ARG	0,560878	0,419162	-14%	0,299401	-26%	0,465070	-10%	0,288091	-27%
267 PHE	1113 LEU		0,033932	new	0,230872	23%	0,023287	2%	0,629408	63%
267 PHE	1114 GLY				0,055888	6%	0,017964	2%	0,113107	11%
267 PHE	1115 ILE	0,932136	0,888889	-4%	0,836993	-10%	0,898869	-3%	0,819028	-11%
267 PHE	1134 GLY								0,226879	new
267 PHE	1188 ARG								0,474385	new
267 PHE	1189 ALA								0,271457	new
267 PHE	1192 ARG								0,640053	new
268 GLY	1110 ARG	0,315369	0,255489	-6%	0,133067	-18%	0,146374	-17%	0,044578	-27%
269 GLY	1136 ASN								0,622754	62%

Hydrogen bonds	life	391,839	534,687	36%	482,332	23%	679,153	73%	465,678	19%
	<N>	1,417	1,340	-5%	1,154	-19%	1,304	-8%	2,215	56%
	DG	-19,685	-20,864	6%	-20,177	3%	-21,062	7%	-19,888	1%

	WT		G185V		G830V		F978A		delF335	
ICH3	NBD2	Frequency	Frequency	Variation	Frequency	Variation	Frequency	Variation	Frequency	Variation
799 GLN	1086 PHE	0,281437	0,292082	1,06%	0,329341	4,79%	0,371257	8,98%	0,125749	-15,57%
800 ASP	1086 PHE	0,734531	0,762475	2,79%	0,774451	3,99%	0,805057	7,05%	0,810379	7,58%
800 ASP	1087 TYR	0,992016	0,990685	-0,13%	0,957419	-3,46%	0,997339	0,53%	0,968729	-2,33%
801 VAL	1044 TYR		0,015968	new	0,124418	12,44%	0,036593	3,66%	0,066533	6,65%
801 VAL	1086 PHE	0,920160	0,868929	-5,12%	0,860279	-5,99%	0,850965	-6,92%	0,887559	-3,26%
801 VAL	1087 TYR	0,560878	0,319361	-24,15%	0,155689	-40,52%	0,214903	-34,60%	0,090486	-47,04%
802 SER	1087 TYR	0,704591	0,473719	-23,09%	0,360612	-34,40%	0,357286	-34,73%	0,234864	-46,97%
805 ASP	1044 TYR	0,908184	0,692615	-21,56%	0,576846	-33,13%	0,629408	-27,88%	0,471723	-43,65%
805 ASP	1045 PRO	0,069860	0,025283	-4,46%	0,019960	-4,99%	0,049900	-2,00%	0,031271	-3,86%
805 ASP	1046 THR	1,000000	0,899534	-10,05%	0,962741	-3,73%	0,996008	-0,40%	0,997339	-0,27%
805 ASP	1047 ARG	0,097804			0,051231	-4,66%	0,441118	34,33%	0,472388	37,46%

Hydrogen bonds	life	533,367	954,057	78,87%	835,569	56,66%	512,837	-3,85%	389,749	-26,93%
	<N>	2,625	2,308	-12,08%	2,673	1,83%	2,623	-0,08%	2,650	0,94%
	DG	-20,461	-21,634	5,73%	-21,322	4,21%	-6,755	-66,99%	-19,667	-3,88%

The variation percentage is estimated based on the mean frequencies of the three replica systems for each P-gp variant against the WT.

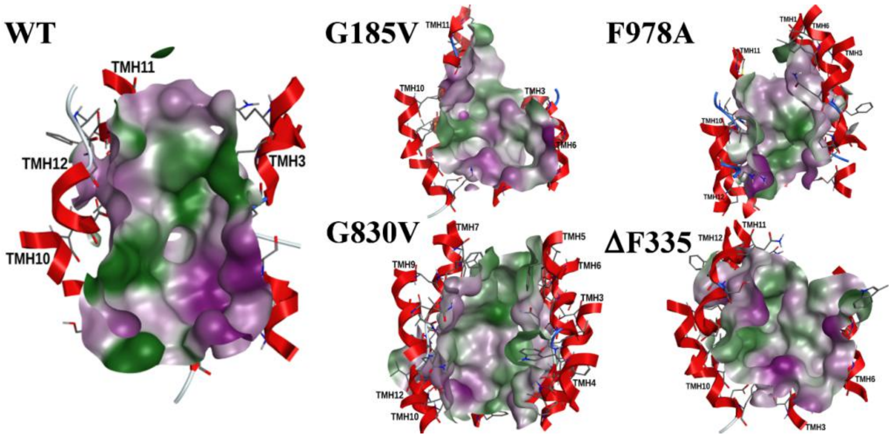

Figure S23. Graphical representation of the molecular surface for the H-site in the human WT P-gp model and variants. Polar regions are defined as pink, hydrophobic regions are colored green.

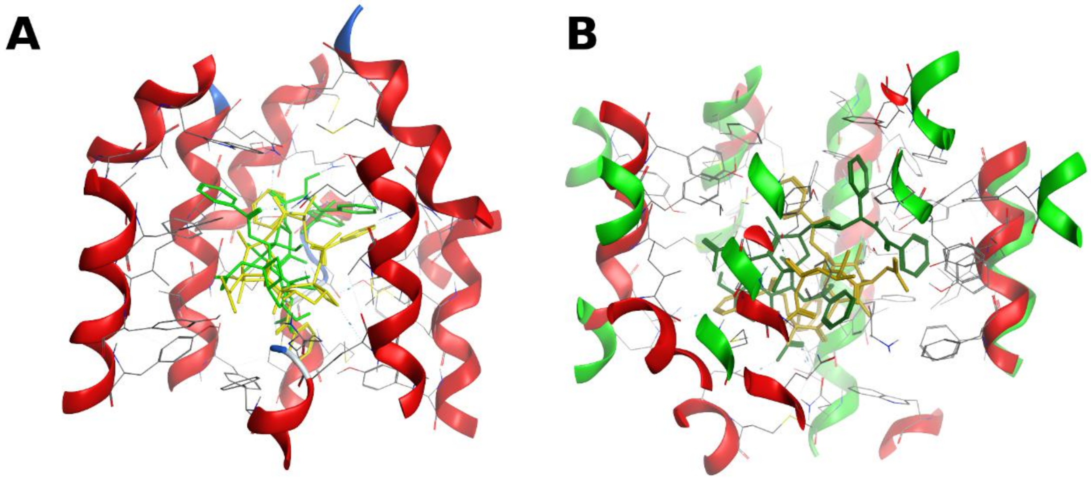

Figure S24. Docking results for taxol molecule in the 6QEX (green, top-ranked pose; yellow, cryo-EM taxol molecule) and in 6QEX (red) and homology (green) models (green, top-ranked pose; yellow, cryo-EM taxol molecule) models.

SUPPORTING PDB FILE

**Name**	all_models.pdb
**Chain ID**	A, WT
	B, G185V
	C, G830V
	D, F978A
	E, ΔF335
